# ATM knock out alters calcium signalling and augments contraction in skeletal muscle cells differentiated from human urine-derived stem cells

**DOI:** 10.1038/s41420-025-02485-x

**Published:** 2025-04-15

**Authors:** Giulia Dematteis, Giulia Lecchi, Giulia Boni, Diana Pendin, Carla Distasi, Mariagrazia Grilli, Dmitry Lim, Luigia Grazia Fresu, Maria Talmon

**Affiliations:** 1 Department of Pharmaceutical Sciences, Università del Piemonte Orientale, Novara, Italy; 2 Department of Health Sciences, School of Medicine, Università del Piemonte Orientale, Novara, Italy; 3 Neuroscience Institute, Padua Section, National Research Council, Padua, Italy

**Keywords:** Stem-cell differentiation, Mechanisms of disease

## Abstract

Ataxia-telangiectasia (A-T) is a rare neurodegenerative disorder caused by the deficiency of the serine/threonine kinase ataxia telangiectasia mutated (ATM) protein, whose loss of function leads to altered cell cycle, apoptosis, oxidative stress balance and DNA repair after damage. The clinical manifestations are multisystemic, among them cerebellar degeneration and muscular ataxia. The molecular mechanism by which ATM loss leads to A-T is still uncertain and, currently only symptomatic treatments are available. In this study, we generated a functional skeletal muscle cell model that recapitulates A-T and highlights the role of ATM in calcium signalling and muscle contraction. To this aim, by using CRISPR/Cas9 technology, we knocked out the ATM protein in urine-derived stem cells (USCs) from healthy donors. The resulting USCs-ATM-KO maintained stemness but showed G2/S cell cycle progression and an inability to repair DNA after UV damage. Moreover, they showed increased cytosolic calcium release after ATP stimulation to the detriment of the mitochondria. The alterations of calcium homoeostasis were maintained after differentiation of USCs-ATM-KO into skeletal muscle cells (USC-SkMCs) and correlated with impaired cell contraction. Indeed, USC-SkMCs-ATM-KO contraction kinetics were dramatically accelerated compared to control cells. These results highlight the relevant function of ATM in skeletal muscle, which is not only dependent on a non-functional neuronal communication, paving the way for future studies on a muscular interpretation of A-T ataxia.

## Introduction

Ataxia-telangiectasia (A-T) is a neurodegenerative disorder resulting from the loss of function of ataxia telangiectasia mutated (ATM) protein [1–3], a serine/threonine kinase involved in the DNA-damage signalling [4]. In particular, in response to DNA double-strand breaks, ATM undergoes trans-autophosphorylation resulting in the switch from a dimeric inactive protein complex to the active monomeric kinase [5, 6], ruling cell-cycle arrest, DNA repair and apoptosis. Some of the ATM substrates, such as p53, have multiple cellular roles and their phosphorylation can lead to cell-cycle arrest or apoptosis depending on the cell type and/or damage level [4, 7]. Cell-cycle arrest is known to be instrumental to prevent replication of damaged DNA and to allow repair, while the apoptosis may be essential to ensure genomic integrity in developing tissues when DNA damage involves progenitor cells [7]. Among several inducing factors of apoptosis, Reactive Oxygen Species (ROS) accumulation is one of the most critical parameters for apoptotic pathway activation, especially within the mitochondria. Different groups have described a ROS-sensing role for ATM which is crucial for maintaining cellular redox homoeostasis inside the mitochondria. Indeed, a ROS increase may drive the opening of the permeability transition pore [8–10], whereas ATM deficiency leads to the generation of mitochondrial ROS [10–12]. Although the mechanisms by which ATM regulates different cellular processes are well characterized, the exact mechanism(s) leading to A-T onset are not fully defined. It is well established that the overall ATM activity is fundamental for preventing neurodegeneration and cellular damage, whereas its loss leads to an altered cellular homoeostasis resulting in the clinical manifestation of a multisystemic disorder. Indeed A-T phenotype is prominently characterized by progressive cerebellar neurodegeneration, accompanied by motor dysfunction (ataxia), ocular and cutaneous telangiectasia, immunodeficiency, gonadal atrophy, radiosensitivity and cancer susceptibility [13], and the mechanisms driving such a dramatic manifestation are still not fully described. Some recent studies hypothesized a non-nuclear function for ATM, for example a role for ATM in stress-induced calcium signalling [14], that can be detrimental for skeletal muscle activity since for example the contractile activity is dependent on calcium release into the cytosol [15]. In particular, in the skeletal muscle, neurotransmitters (e.g. acetylcholine) lead to Ca^2+^ release from the store into the cytosol via ryanodine receptors channels, which in turn activates the contractile apparatus [15]. Thus, given the key role of Ca^2+^ in muscle physiology, and considering that alterations of Ca^2+^ homoeostasis are responsible in many muscular disorders, investigating the ATM role in calcium signalling could increase the knowledge on A-T pathophysiology [12, 13]. In fact, the muscular phenotype of A-T has been interpreted as secondary to innervation dysfunctions and is still considered as such [16–19] while it would be interesting to evaluate if and how the absence of ATM in skeletal muscle can directly impact muscle functionality by influencing calcium homoeostasis. Considering that A-T, as well as many muscular disorders, is a rare disease, there is an urgent need to develop robust, and easy-to-manage cell models that recapitulates cellular dysfunction due to the ATM loss, which would be useful for the investigation of molecular A-T pathogenesis and for drug screening and/or repurposing. Urine-derived stem cells (USCs) have recently emerged as a promising source of adult stem cells for disease modelling, drug screening, and precision medicine because endowed of remarkable ability to differentiate into several cell types [20–22]. In particular, we already characterized skeletal muscle cells (SkMCs) derived from USCs demonstrating a functional calcium machinery and the acquisition of an excitable cellular phenotype capable of contracting following different stimuli [23, 24]. Taking advantage of this well-described skeletal muscle cell model, in the present work we generated an A-T cellular model using USCs knocked out for ATM by CRISPR/Cas9 technology and differentiated them into skeletal muscle cells providing a useful platform for studying the molecular mechanism underlying the ataxia-telangiectasia phenotype. Specifically, we demonstrated that USCs-ATM-KO recapitulate ATM-associated dysfunction reported in the literature, but also provide a valuable model to investigate non-neuronal functions of ATM. Indeed, USCs-ATM-KO displayed altered intracellular calcium signalling and reduced mitochondrial ATP-related respiration, while SkMCs-ATM-KO showed an altered calcium machinery pattern and consequently faster contraction kinetic.

## Results

### USCs knock-out for ATM retain stem cell properties

**Fig. 1:**
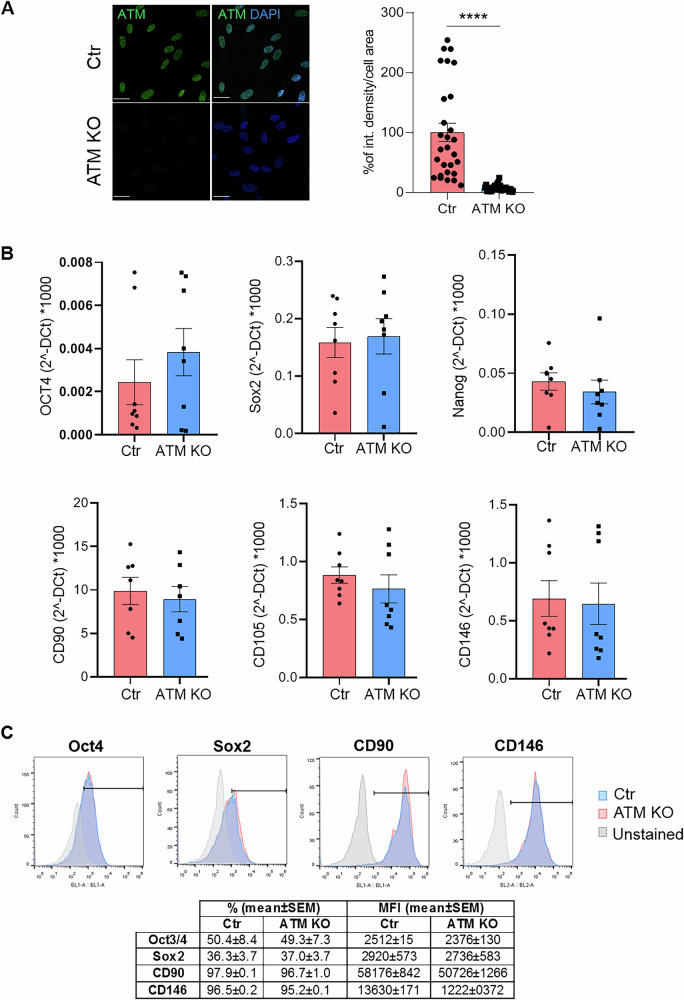
Characterization of USCs-ATM-KO. **A** Representative IF analysis of ATM expression in non-transfected USCs-Ctr and USCs-ATM-KO (Magnification 63X; scale bar= 20 µm; Green: ATM; Blue: DAPI) and relative fluorescence quantification. Data are expressed as % of integrated density/cell area and are the mean ± SEM of 28 cells of 3 independent experiments. **B** qPCR analysis of stem (Oct4, Sox2, Nanog) and mesenchymal (CD90, CD105, CD146) markers. Data are mean ± SEM of 8 independent experiments. **C** FACS analysis of Oct4, Sox2 CD90, and CD146. Data are shown as representative superimposed histograms and, in table, as mean ± SEM of at 4 independent experiments.

To generate an A-T cellular model starting from USCs, CRISPR/Cas9 technology was applied *via* transfection of a specific construct targeting the ATM gene. To validate the KO of ATM in USCs (USCs-ATM-KO), immunocytochemical analysis was performed. As shown in Fig. 1A, characteristic nuclear localization of ATM is present in USCs-Ctr, whereas no signal was detected in KO (Fig. 1A). The KO of ATM protein was also confirmed by Western blot analysis (Supplementary Fig. 1). Remarkably, the suppression of ATM protein did not affect the stemness properties of USCs as demonstrated by the still present expression of stem cell (Oct4, Sox2, Nanog) and mesenchymal stem cells (CD90, CD105, CD146) markers both at RNA (Fig. 1B) and protein level (Fig. 1C).

### USCs-ATM-KO showed altered cell cycle, apoptosis and mitochondrial dysfunction

**Fig. 2:**
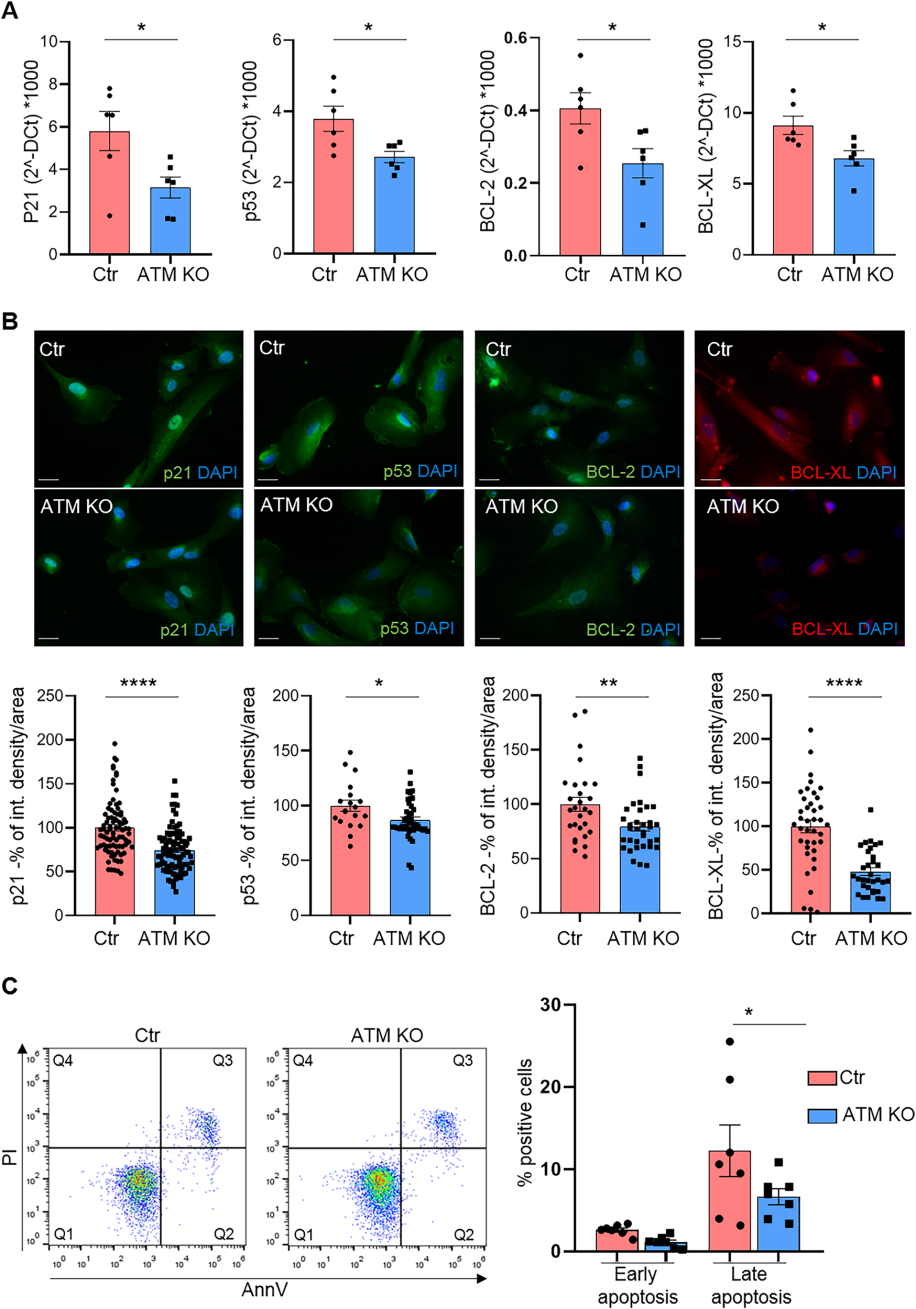
USCs-ATM-KO altered apoptosis. **A** Real-time PCR analysis of apoptosis-related genes. Data are expressed as 2-ΔCt*1000 and are mean ± SEM of 6 independent experiments. *p < 0.05 vs USCs-Ctr. **B** Representative IF analysis of apoptosis-related markers expression in USCs-Ctr and USCs-ATM-KO (Magnification 40X; scale bar= 25 μm) and relative fluorescence quantification. Data are expressed as % of integrated density/cell area and are the mean ± SEM of cells from 3 independent experiments. **C** FACS analysis of apoptosis. Cells were stained with Annexin V and propidium iodide (PI) and the fluorescence was evaluated by cytofluorimeter. Data are shown by representative dot plots and mean ± SEM of % of cells in early (Q2) and late (Q3) apoptosis (*n* = 7). *p < 0.05 vs USCs-Ctr.

**Fig. 3:**
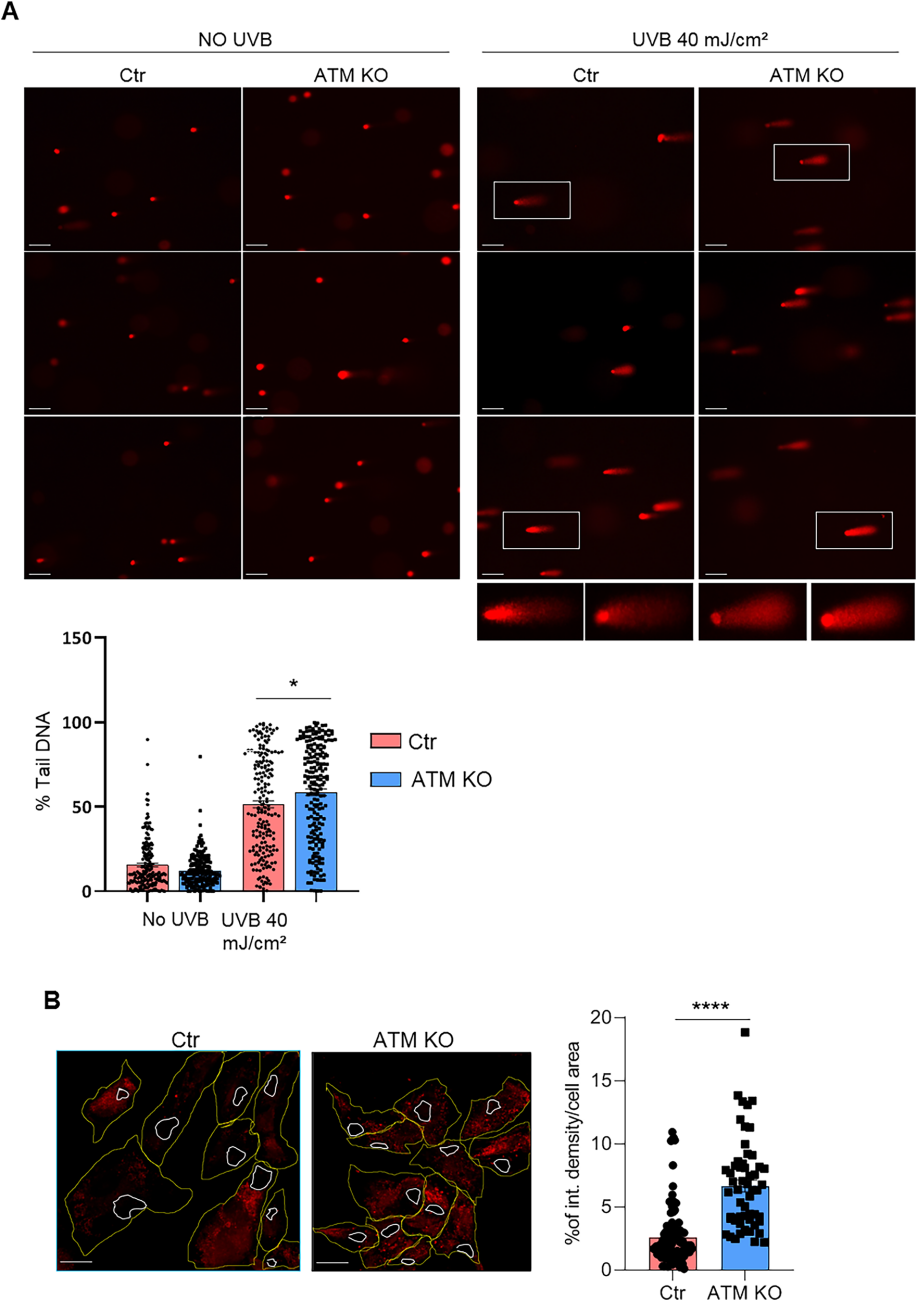
USCs-ATM-KO DNA damage repair and mitochondrial oxidative stress. **A** Representative images and quantification of comet assay analysis (Magnification 20X; insets zoom 2.5X; scale bar = 100 µm). USCs-Ctr and USCs-ATM-KO were stimulated with UVB 40 mJ/cm^2^ and, after 6 h of recovery, stained with PI and separated by electrophoresis. Data are expressed as mean ± SEM of % tail DNA of at least 176 cells in 3 independent experiments. *p < 0.05 vs UVB-stimulated USCs-Ctr. **B** Representative confocal images (40X magnification objective; scale bar = 30 µm) and fluorescence quantification of mitoSOX staining in USCs-Ctr and USCs-ATM-KO. The outline of the area of the cell is depicted in yellow, and that of the nuclei in white. Data are expressed as % of integrated density/cell area and are the mean ± SEM of 92 Ctr and 56 ATM-KO cells from 3 independent experiments.

We then analysed and compared cell cycle, apoptosis and oxidative stress in both USCs-Ctr and the KO cells and we found that USCs-ATM-KO displayed altered proliferation rate (Supplementary Fig. 2A), cell cycle (Supplementary Fig. 2B and C), apoptosis (Fig. 2A–C) and DNA repair capability (Fig. 3A), faithfully recapitulating the phenotype of ATM-defective cells. Indeed, we found a reduction, at 24, 48 and 96 h after seeding, in the proliferation rate of USCs-ATM-KO (Supplementary Fig. 2A). The percentage of USCs-ATM-KO in G2/S phases was higher compared to USCs-Ctr and slightly decreased in G1 and SubG1 phases (Supplementary Fig. 2B). This trend was increased after UVB stimulation (Supplementary Fig. 2C) suggesting that USCs-ATM-KO were unable to stop growing after receiving a DNA insult, resulting in DNA mutation accumulation. Moreover, we observed that, as expected, apoptosis is affected by the deletion of ATM. In fact, the expression of both genes (Fig. 2A) and proteins (Fig. 2B) involved in apoptosis regulation is significantly altered, as demonstrated by qPCR and IF analysis of p21, p53, BCL-2 and BCL-XL (Fig. 2A, B), among which p53 and p21 are responsible for G1 arrest after DNA damage, thus their downregulation is consistent with the altered cell cycle. As further confirmation a functional assay was performed by AnnexinV/propidium iodide (PI) cytofluorimetric analysis and we have observed a significant decrease in the percentage of cells in late apoptosis (Fig. 2C). By single-cell electrophoresis analysis of both USC populations after UVB challenge (40 mJ/cm^2^; Fig. 2C) we strongly corroborated the hypothesis of mutation accumulation linked to the cell cycle deregulation. In fact, comet assay analysis revealed a significantly higher percentage of tail DNA in USC-ATM-KO than the USC-Ctr (Fig. 3A), even after 6 hours of recovery (Fig. 3A) confirming the role of ATM in the repair of DNA damage induced by UVB stimulation (Fig. 3A). Finally, among the different functions of ATM, Xie and collaborators [9] have shown that it could play a role as a sensor and regulator of ROS production in mitochondria. Therefore, when evaluating ROS levels in mitochondria by MitoSOX staining, we observed a significant increase in the probe signal in USCs-ATM-KO compared to USC-Ctr cells (Fig. 3B).

### USCs-ATM-KO showed altered calcium homoeostasis

**Fig. 4:**
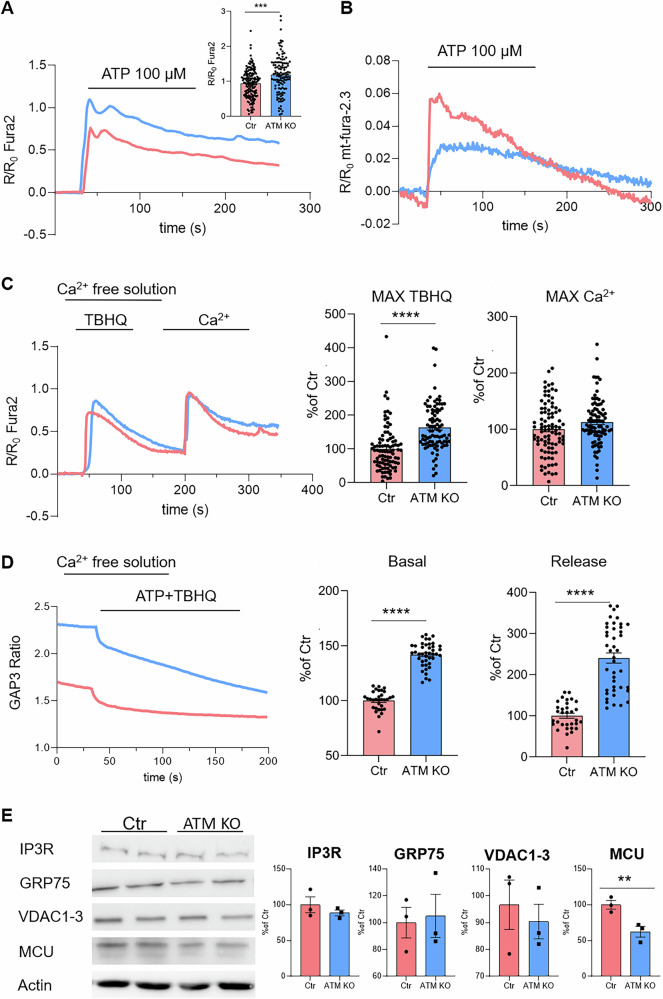
USCs-ATM-KO display deregulated calcium homoeostasis. **A** ATP-induced (100 μM) cytosolic Ca^2+^-release in USCs-Ctr and USCs-ATM-KO. Data are illustrated as representative traces as well as histograms (inset) of mean ± SEM of maximum peaks (ΔR/R_0_ Fura2) of 151 Ctr and 118 ATM-KO cells from 3 independent experiments. **B** ATP-induced (100 μM) mitochondrial Ca^2+^-signalling in USCs-Ctr and USCs-ATM-KO. Data are illustrated as representative traces as well as histograms (inset) of mean ± SEM of maximum peaks of maximum peaks (ΔR/R_0_ mt-fura-2.3) of 118 Ctr and 115 ATM-KO cells from 3 independent experiments. **C** Representative traces of Ca^2+^-release induced by TBHQ (50 μM) and subsequent SOCE. Histograms of mean ± SEM of maximum peaks following TBHQ stimulus and Ca^2+^ (2 mM) addition (ΔR/R_0_ Fura2) of 92 cells from 3 independent experiments. **D** Representative traces and histograms of mean ± SEM of maximum peaks of calcium analysis (basal calcium and ER calcium release) of cells (*n* = 40 cells) transfected with GAP3-ER Calcium indicator. **E** Representative Western blot and densitometric analysis of IP3R, GRP75, VDAC1-3 and MCU expression in USCs-Ctr and USCs-ATM-KO. Data are mean ± SEM (*n* = 4 independent experiments) of the % of the Ctr. **p < 0.01 USCs-ATM-KO vs USCs-Ctr; ***p < 0.001 USCs-ATM-KO vs USCs-Ctr; ****p < 0.0001 USCs-ATM-KO vs USCs-Ctr.

To evaluate whether ATM loss could affect calcium signalling, we measured the cytosolic, mitochondrial and endoplasmic reticulum (ER) Ca^2+^ dynamics following stimulation with a purinergic agonist ATP (Fig. 4A–D) and the expression of calcium signalling toolkit components (Fig. 4E) in both USCs-Ctr and USCs-ATM-KO. Firstly, Fura-2-loaded USCs-ATM-KO, challenged with ATP in the presence of external calcium, had significantly higher calcium transients in the cytosol compared to USCs-Ctr cells (Fig. 4A). Contemporary, we showed that ATP-induced Ca^2+^ transients in the mitochondrial matrix were significantly lower in mt-fura-2.3-loaded USCs-ATM-KO compared to USCs-Ctr (Fig. 4B). We therefore assessed cytosolic calcium entry by challenging cells with a SERCA (sarco-endoplasmic reticulum calcium ATPase) poison, TBHQ, to induce ER calcium depletion in a calcium-free medium. Store-operated calcium Entry (SOCE) was then assessed by re-addition of Ca^2+^ to the external medium. Following this protocol, as described in Fig. 3C, we observed an increased efflux of Ca^2+^ from the ER in USCs-ATM-KO, while no differences in SOCE were found (Fig. 4C). This suggests that an increased cytosolic Ca^2+^ signalling may be mediated by an increase in the releasable ER Ca^2+^ pool. To further corroborate this interpretation, we transfected USCs with a construct expressing ER lumen-targeted GAP3 probe [25], which allows the recording of calcium levels in the ER lumen. We observed that USCs-ATM-KO have a higher steady-state Ca^2+^ content than the USCs-Ctr (Fig. 4D) and release more Ca^2+^ from the ER after combined stimulus with TBHQ and ATP (Fig. 4D). The results obtained on ER and mitochondrial Ca^2+^ transients, correlate with the reported observation that ER Ca^2+^ storage and release regulate mitochondrial Ca^2+^ uptake into the mitochondrial matrix via a direct Ca^2+^ transfer from the ER to mitochondria at the ER-mitochondria contact sites by a complex formed by IP3R, VDAC1 and GRP75; we also assessed mitochondria calcium uptake upon ATP stimulation by mitochondrial matrix-targeted Fura-2 variant (mt-fura-2.3) [26, 27]. Indeed, the reduced ATP-induced Ca^2+^ transients in the mitochondrial matrix in USCs-ATM-KO is probably due to the significantly reduced MCU expression level, while no differences were observed for IP3R, GRP75 and VDAC1/3 (Fig. 4E).

### USCs-ATM-KO can be efficiently differentiated into skeletal muscle cells

**Fig. 5:**
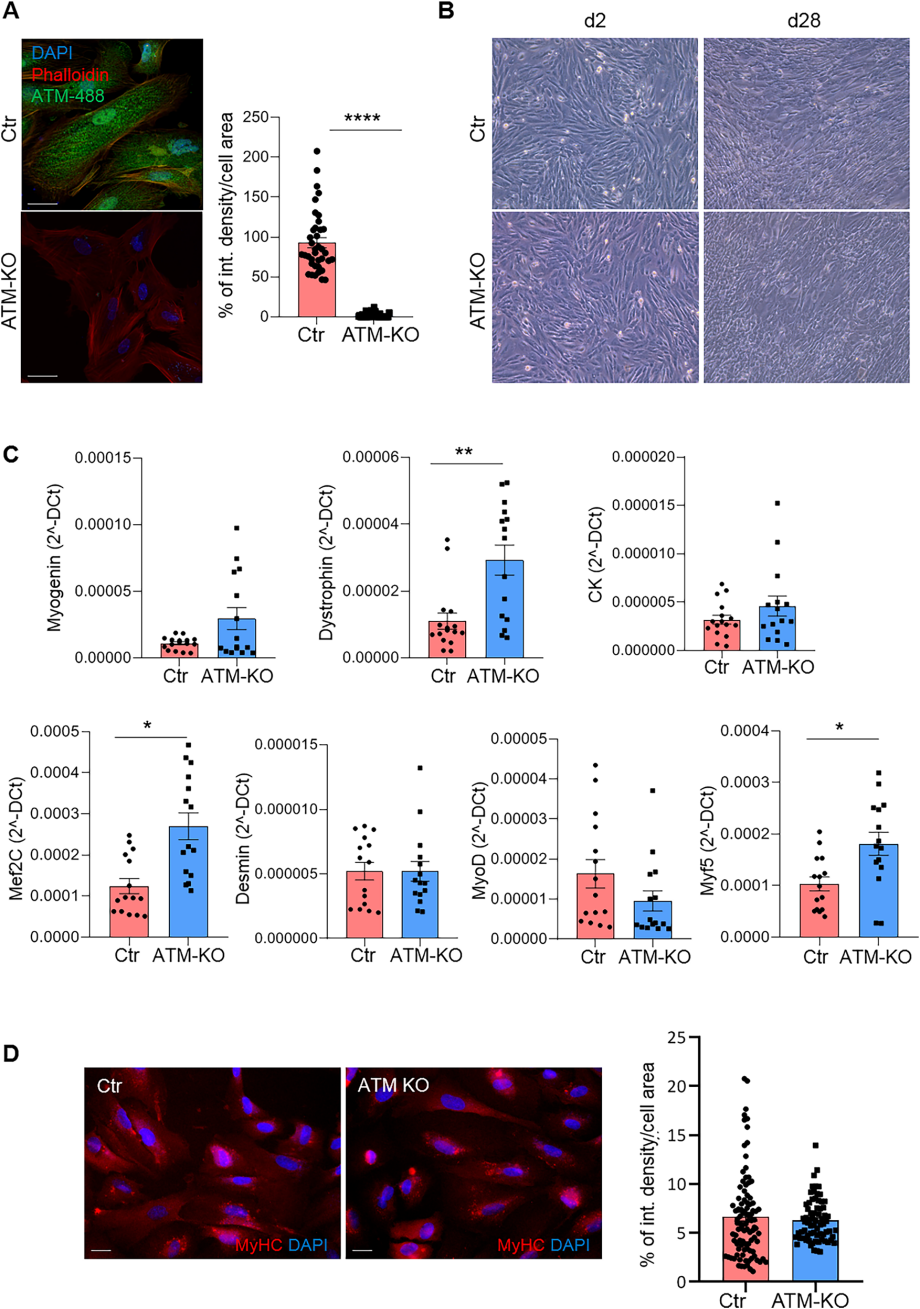
USC-SkMCs-ATM-KO characterization. **A** Representative confocal images of ATM expression in USC-SkMCs-Ctr and ATM-KO (Magnification 63X; scale bar= 25 µm; Green: ATM; Red: Phalloidin; Blue: DAPI) and relative fluorescence quantification. Data are expressed as % of integrated density/cell area and are the mean ± SEM of 38 Ctr and 35 ATM-KO cells from 3 independent experiments. ****p < 0.0001 USCs-ATM-KO vs USCs-Ctr. **B** Representative phase-contrast images of USCs-Ctr and USCs-ATM-KO at different stages of differentiation towards SkMCs (d2, d14 and d28; magnification 200x). **C** qPCR analysis of skeletal muscle cell markers (myogenin, dystrophin, creatine kinase-CK, Mef2C, desmin, MyoD, Myf5). Data are mean ± SEM of at 15 independent experiments. *p < 0.05 and **p < 0.01 USC-SkMCs-ATM-KO vs Ctr (**D**) Representative confocal images of MyHC expression in USC-SkMCs-Ctr and ATM-KO (Magnification 40X; Red: MyHC; Blue: DAPI) and relative fluorescence quantification. Data are expressed as % of integrated density/cell area and are the mean ± SEM of 97 Ctr and 71 ATM-KO cells from 3 independent experiments.

Taking advantage of a well-characterized USCs differentiation protocol *via* MyoD induction, we then moved to explore the impact of ATM deficiency on skeletal muscle cells (USC-SkMCs) derived from both USCs-Ctr and USCs-ATM-KO. First of all, we assessed whether the ATM-KO was retained during the differentiation process, as shown by immunofluorescence staining and Western blot analysis (Fig. 5A and Supplementary Fig. 3). Of note, the differentiation process results in cytoplasmic localization of ATM protein in line with published reports [28, 29] (Fig. 5A). It is possible to observe that the absence of ATM has not compromised the differentiation of the USCs and that the SkMCs derived from USCs-Ctr and USCs-ATM-KO are morphologically similar (Fig. 5B) with a comparable expression of early (e.g. MyoD and desmin) and mature (e.g. dystrophin and creatine kinase) myogenic markers (Fig. 5B). However, transcription factors involved in early myogenesis and muscle regeneration (e.g. Mef2C and Myf5) are expressed at higher levels in USC-SkMCs-ATM-KO compared to Ctr (Fig. 5C). The immunofluorescence analysis of MyHC showed a comparable protein level of expression (Fig. 5D).

### USC-SkMCs-ATM-KO showed altered calcium homoeostasis

**Fig. 6:**
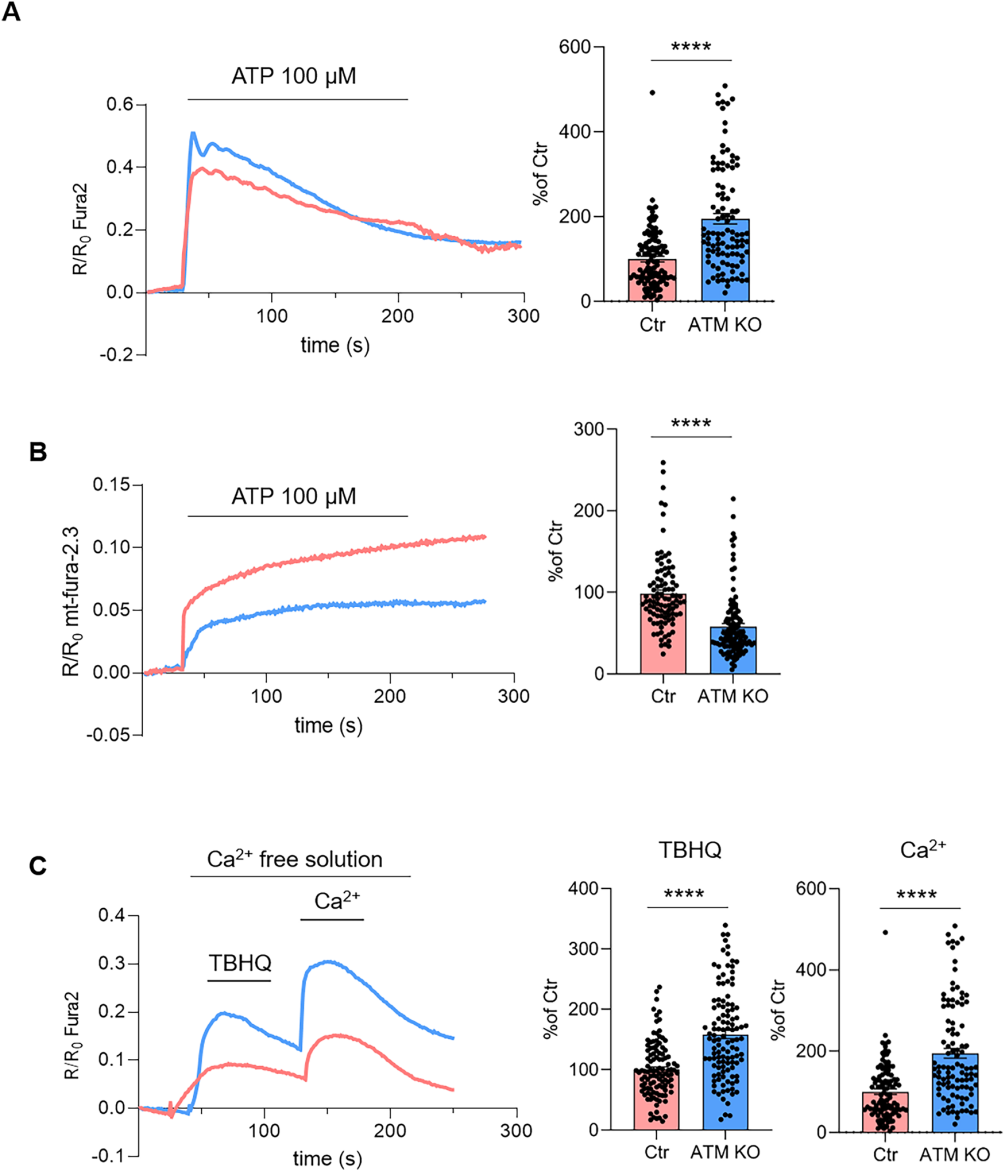
USC-SkMCs-ATM-KO calcium homoeostasis. **A** ATP-induced (100 μM) cytosolic Ca^2+^-release in USC-SkMCs-Ctr and ATM-KO. Data are illustrated as representative traces as well as histograms of mean ± SEM of maximum peaks (ΔR/R_0_ Fura2) of 180 Ctr and 119 ATM-KO cells from 3 independent experiments. **B** ATP-induced (100 μM) mitochondrial Ca^2+^-signalling in USC-SkMCs-Ctr and ATM-KO. Data are illustrated as representative traces as well as histograms of mean ± SEM of maximum peaks (ΔR/R_0_ mt-fura-2.3) of 97 Ctr and 111 ATM-KO cells from 3 independent experiments. **C** Representative traces Ca^2+^-release induced by TBHQ (50 μM) and subsequent SOCE. Histograms of mean ± SEM of maximum peaks following TBHQ stimulus and Ca^2+^ (2 mM) addition (ΔR/R_0_ Fura2) of 112 cells from 3 independent experiments. ****p < 0.0001 vs Ctr.

**Fig. 7:**
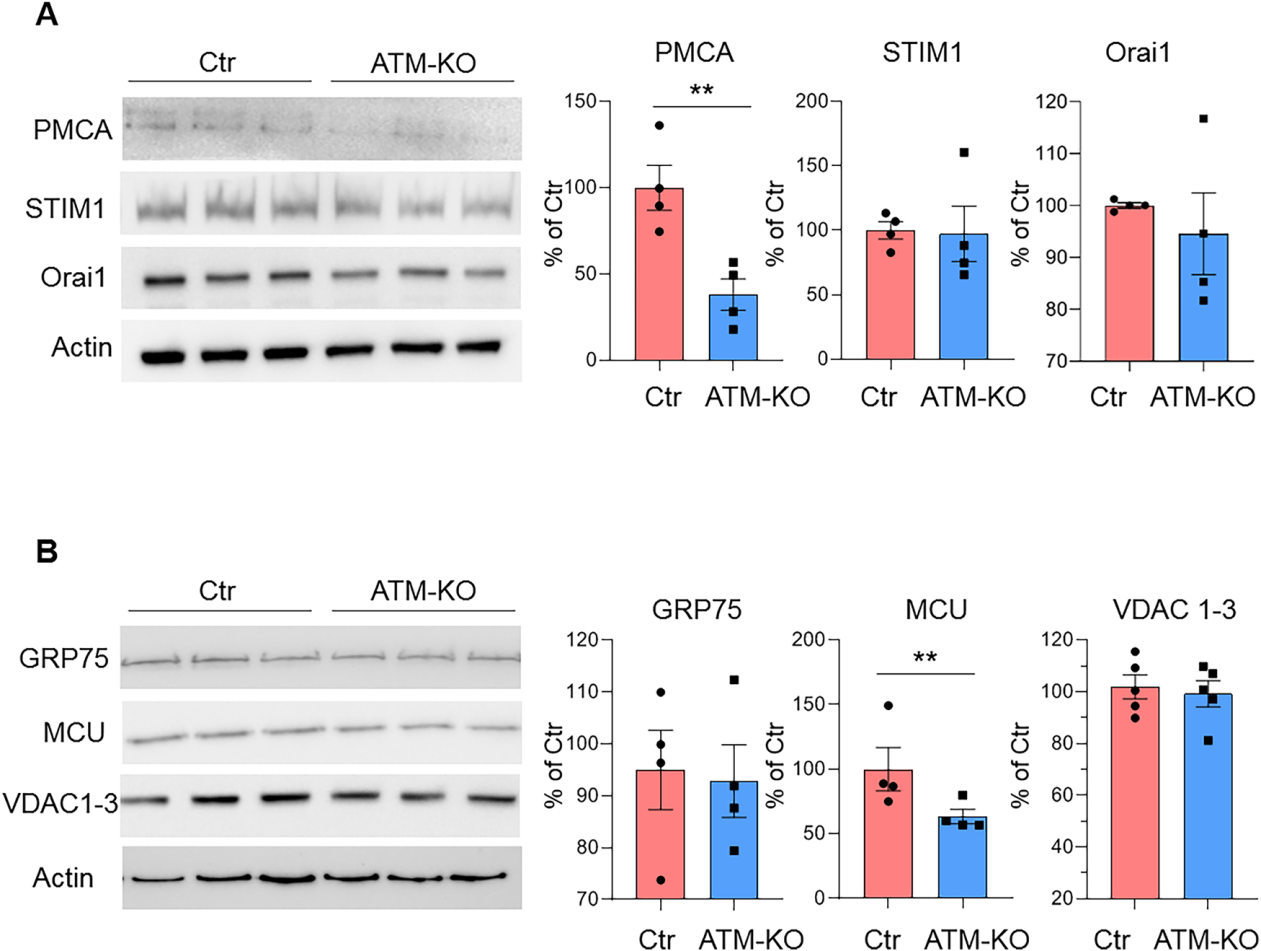
Calcium players expression in USC-SkMCs-ATM-KO. Representative Western blots and densitometric analysis of **A**) PMCA, STIM1, Orai1 and **B**) GPR75, MCU, VDAC1-3 expression in skeletal muscle cells derived from both USCs-Ctr (SkMCs-Ctr) and ATM-KO (SkMCs-ATM-KO). Data are mean ± SEM (*n* = 4 independent experiments) of the % of the Ctr. *p < 0.05 and **p < 0.01 vs Ctr.

At this point it was important to compare USC-SkMCs-Ctr and USC-SkMCs-ATM-KO from a functional point of view. Live imaging of Ca^2+^ transients in the cytosol and mitochondria confirmed that USC-SkMCs-ATM-KO had higher cytosolic Ca^2+^ transients after ATP stimulation (Fig. 6A), while mitochondrial calcium uptake was impaired (Fig. 6B), similar to parental USCs. We then evaluated cell response in calcium-free medium to SERCA block by TBHQ, and subsequent Ca^2+^ re-addition (Fig. 6C). USC-SkMCs-ATM-KO showed a more pronounced SOCE in comparison to USC-SkMCs-Ctr (Fig. 6C) as demonstrated by the significantly augmented average calcium peaks in USC-SkMCs-ATM-KO following both TBHQ and external calcium challenges (Fig. 6C). These data suggest an increase in SOCE activity, even though both STIM1 and Orai1 protein levels are not affected by ATM-KO (Fig. 7A). Moreover, protein expression analysis demonstrated that Plasma Membrane Calcium ATPase (PMCA) (Fig. 7A) and the MCU (Fig. 7B) were significantly reduced in USC-SkMCs-ATM-KO while neither GRP75 nor VDAC1-3 protein levels were affected (Fig. 7B).

### USC-SkMCs-ATM-KO showed impaired contraction kinetic

**Fig. 8:**
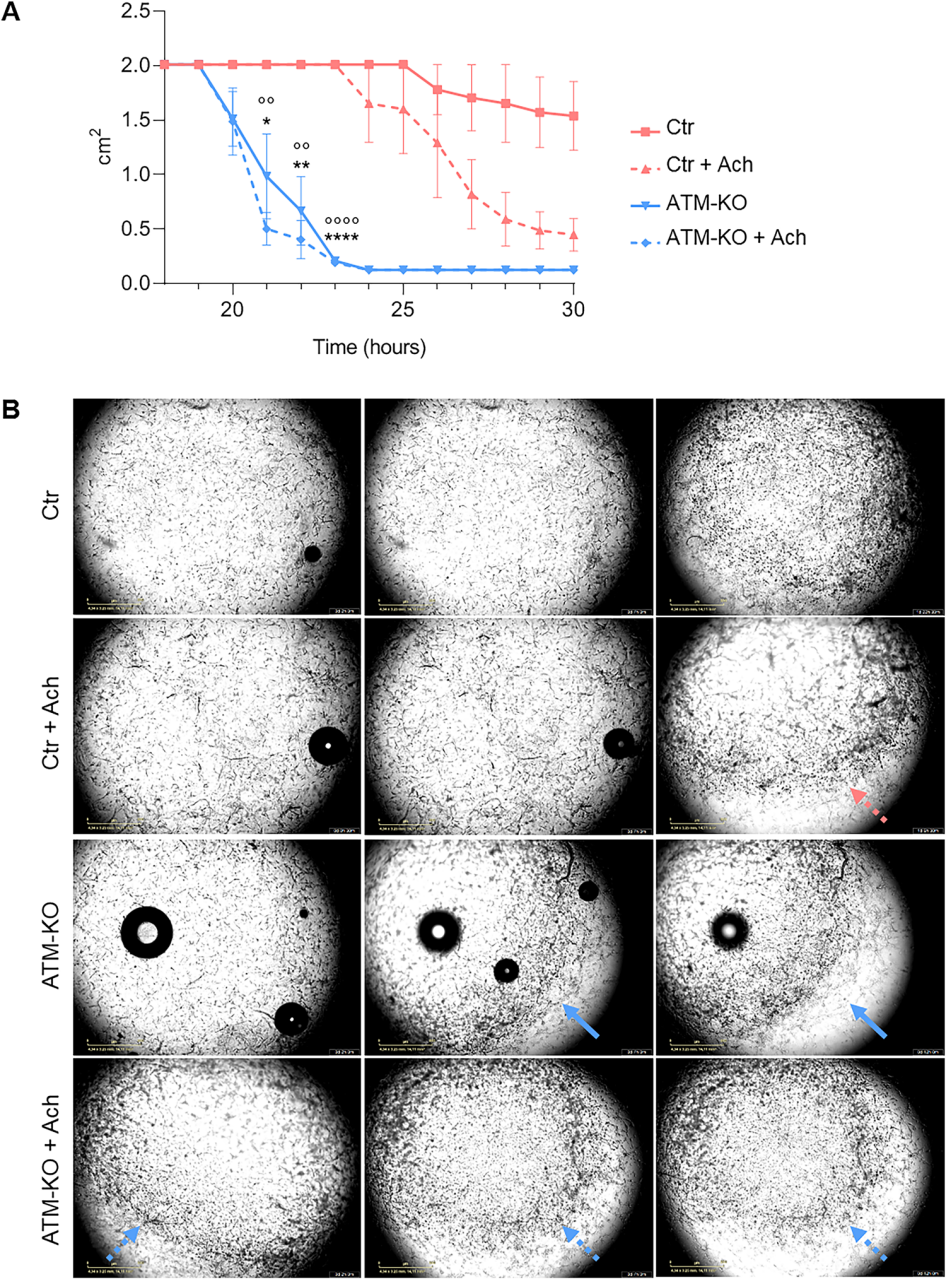
Collagen contraction assay. USC-SkMCs were plated on collagen discs and treated with acetylcholine (100) μM. **A** The collagen area was measured at the indicated time points. Data are mean ± SEM of collagen area (cm^2^) at several time points (*n* = 3) *p < 0.05, **p < 0.01, ****p < 0.0001 USC-SkMCs-ATM-KO vs Ctr; °°*p* < 0.01, °°°°*p* < 0.0001 USC-SkMCs-ATM-KO + Ach vs Ctr + Ach. **B** Representative phase-contrast images of collagen discs at progressive time points. The arrow (pink for control and blue for the ATM-KO) indicates the reduction of the collagen area.

Since ATM deficiency affects calcium homoeostasis in skeletal muscle cells, we evaluated if it consequently affects the calcium-dependent contractile activity USC-SkMCs-Ctr and USC-SkMCs-ATM-KO. Cells were embedded into collagen discs and then stimulated with acetylcholine (Ach). The contraction of muscle cells results in a reduction of the diameter of the collagen discs, which can be quantified. We observed a dramatic difference in kinetics between USC-SkMCs-ATM-KO and USC-SkMCs-Ctr (Fig. 8). Indeed, both unstimulated and treated SkMCs-ATM-KO started to contract significantly earlier compared to the USC-SkMCs-Ctr, as showed by both the average traces (Fig. 8A) and the representative phase-contrast images (Fig. 8B). Indeed, the contraction of USC-SkMCs-ATM-KO started around 20 h after the seeding, about 4 h earlier than USCs-Ctr. Treatment with acetylcholine induced a faster but less pronounced contraction in cells lacking ATM protein (Fig. 8A, B).

## Discussion

This work was designed to assess i) whether a valid A-T model, useful for drug development and repurposing, can be generated from human USCs, and ii) whether differentiated USC-SkMCs-ATM-KO retained A-T phenotype and may be useful for investigating mechanisms associated with muscle dysfunction in A-T.

Our data suggest that ATM deficiency in USCs did not affect pluripotency and stem cell markers expression. The resulting cellular model effectively recapitulates cellular dysfunctions caused by ATM deficiency in both DNA damage-dependent and ROS-dependent activation, confirming the canonical role of ATM in regulating DNA repair, cell cycle arrest and, in case, autophagy [1]. This is in line with a previous report on iPSCs derived from A-T patients [30]. Specifically, we observed a block of USCs-ATM-KO in G2/S cell cycle phase and apoptosis resistance, which is consistent with reported defective checkpoint activation in A-T iPSCs [30] and in mouse/human embryonic stem cells silenced or inhibited for ATM [31]. One of the most important characteristics of cells derived from A-T patients or different A-T in vitro and mouse models [8, 14, 32–34] is mitochondria instability with higher ROS levels compared to the control. Our USCs-ATM-KO model also showed higher oxidative stress in the mitochondria, confirming mitochondrial dysfunction. Mitochondrial functions strongly rely on calcium signalling. Indeed, mitochondrial calcium levels regulate the activity of both KREB cycle and ATP synthase, as well as the opening of the permeability transition pore and the consequent activation of the apoptotic pathway [35]. In turn, mitochondrial calcium is directly dependent on cytosolic calcium signalling. Therefore, a comprehensive analysis of intracellular calcium signalling in ATM-deficient USCs was essential. We demonstrated that ATM-KO induced a significant increase in cytosolic calcium upon purinergic stimulation, due to increased ER calcium levels and release. Surprisingly, the increase in cytosolic calcium was not followed by an increase in mitochondrial calcium. On the contrary, we found a remarkable reduction in mitochondrial Ca^2+^ uptake. These results may be explained by the reduced expression in ATM-KO cells of MCU (decreased by ≈40% at the protein level), the rate limiting Ca^2+^ transporter, driving the uptake of ions into the mitochondrial matrix.

The obtained skeletal muscle cells starting from USCs-ATM-KO demonstrate that the absence of ATM slows down the differentiation process, since we observed a higher level of expression of the early stage myogenesis transcription factors (Mef2C, Myf5 and, slightly, myogenin) that have to be switched off in mature myocytes. Moreover, the increased dystrophin expression could be interpreted as a compensatory mechanism in response to the calcium deregulation, to the increased oxidative state and to the damages accumulation that reflects the muscle abnormalities observed by Tassinari et al. in an ATM null mouse model [36]. The calcium signalling alterations retained by the USC-SkMCs-ATM-KO are responsible for the direct effect on the intrinsic properties of skeletal muscle cells, such as the dramatically increased contractility. In most patients, limb tremors and incoordination may appear at around 9-10 years of age and progressively worsen together with choreoathetosis leading to muscle weakness and ataxia. Other muscle-associated features have been described in a murine model of A-T and include muscle wasting and atrophy, an altered sarcomere organization, aberrant mitochondrial number and morphology, accompanied by increased ROS production [36]. TEM analysis of myofibers has also revealed ultrastructural defects in both the contractile machinery and organelles involved in producing cellular energy, suggesting an increased oxidative stress response [36]. Canonically, the muscular phenotype of A-T has been interpreted as secondary to innervation dysfunctions [16–19]. In this scenario, an increasing number of reports suggest that ATM deficiency may lead to intrinsic muscle alterations, which may account, at least in part, for A-T-related symptoms. For example, it has been suggested that ATM is indispensable for skeletal muscle protein synthesis through the PI3K/Akt pathway [37, 38]. In line with this hypothesis, herein we demonstrated that ATM loss leads to muscle intrinsic alterations, higher contractile activity, compatible with enhanced cytosolic Ca^2+^ signals in USC-SkMCs-ATM-KO.

USCs are adult renal stem cells that exhibit stemness properties, multi-differentiation potential and self-renewal capability of mesenchymal stem cells [39, 40]. Moreover, they represent a non-invasive, cost-effective and easy-to-manage source of stem cells [20, 41, 42], which is particularly relevant for paediatric A-T patients. USCs can be easily and repeatedly isolated from urine samples, and obtained from donors regardless of age, sex, and health status and without any ethical concerns [42]. They can be cultured to high passages and cryopreserved without loss of viability, proliferation and differentiation potential [40]. They show high degree of stemness, being able to differentiate into several cell types beyond the urinary system including neurons [43, 44] and skeletal muscle cells [23, 45]. Indeed, since their discovery, USCs have been used to model several genetic diseases [41, 46, 47] both as stem cells [48, 49] and after direct reprogramming into differentiated cells [50, 51]. Zhang and colleagues demonstrated that USCs isolated from spinal muscular atrophy (SMA) patients efficiently recapitulate survival motoneuron (SMN) defects and are useful for screening potential compounds for SMA treatment without being terminally differentiated [52], saving time and resources. More recently, RNA-Seq analysis has been performed both on freshly isolated USCs and MyoD-reprogrammed USCs derived from patients affected by several groups of neuromuscular disorders [53], demonstrating that the genetic signature of the disease is present in the USCs and that, importantly, is maintained after USCs differentiation into skeletal muscle cells [53]. In this work we focused on the development of a novel human cellular A-T model platform for basic research and drug development. Finally, we did not use either cells from mouse A-T models or a material from A-T patients, which might be considered as a limitation of this work.

In conclusion, our results suggest that (i) USCs-KO for ATM faithfully recapitulate the key A-T phenotype and, therefore, represent a valuable model for drug development and repurposing in A-T; (ii) SkMCs differentiated from USCs-ATM-KO retain both the muscle phenotype and A-T-specific alterations, suggesting that USC-SkMCs-ATM-KO can be used to study mechanisms of A-T pathogenesis; (iii) muscle-intrinsic alterations of Ca^2+^ handling and contractility may account, at least in part, for muscle related A-T symptoms.

## Materials/Subject and Methods

### Urine-derived stem cells (USCs) isolation and culture

The collection of human urine from healthy volunteers was approved by the local Ethics Committee (Comitato Etico Interaziendale Maggiore della Carità, Novara; authorization CE 190/20), all donors have signed the informed consent, and the work was carried out in accordance with the Declaration of Helsinki. USCs were isolated from urine samples (30–300 ml) collected from 5 healthy individuals (age from 32 to 59 years old). The samples were preserved with 10% primary medium (DMEM/F12, Sigma Aldrich, Cat. #D8062; 10% FBS, Euroclone, Cat. #ECS5000L; 1% penicillin-streptomycin, Euroclone, Cat. #ECB3001; 2.5 μg/ml amphotericin B, GIBCO, Cat. #15290018; Renal Epithelial Growth Medium SingleQuot supplement, LGC Standard, Cat. # ATCC-PCS-400-040) for 1 h at 4 °C before the isolation. USCs were isolated and differentiated as previously described [23]. Briefly, urine samples were centrifuged (10 min, 400 g) and the pellet was washed twice in wash buffer. The obtained cells were plated in a 0.1% gelatin-coated coated-24-well plate in 500 µl of primary medium. 24, 48, and 72 h later 500 µl of primary medium were added. Then, 1.5 ml of medium was removed and 500 µl of proliferation medium was added. Half of the medium was changed daily.

### USCs-ATM-KO generation

To generate the ATM-KO, CRISPR/Cas9 constructs was used (sc-400192, Santa Cruz Biotechnology, Inc.). The commercial CRISPR/Cas9 constructs kit allows the identification and cleavage of the ATM gene, warranting maximum knockout efficiency, moreover this kit ensures the identification of plasmid-receiving cells with a transient expression of GFP, that could be used for the selection of the KO population. 100.000 USCs have been plated in P35 dish. 24 h later, cells at 70% of confluence, were transfected with transfection mix (Lipofectamine Stem Reagent, Invitrogen, Cat. #STEM00003) + DNA in a 1:3 ratio. 24 h post transfection GFP positive cells were sorted (S3e Cell Sorter, Bio-Rad, Segrate, Milano), and plated (1.500 USCs for each well of a 96 MW plate). 48 h later, confluent wells have been expanded, and after 2 passages, have been used for experiments. GFP negative cells were also collected and plated too and used as control (Ctr) for all the experiments.

### USCs differentiation to skeletal muscle cells (USC-SkMCs)

USCs were differentiated into skeletal muscle cells (USC-SkMCs) as previously reported [23]. Sub-confluent USCs, both control and ATM-KO, were transduced with a second-generation lentiviral vector carrying an inducible MyoD insert (LV-TRE-VP64 human MyoD-T2A-dsRedExpress2), that was a gift from Charles Gersbach (Addgene plasmid # 60629; http://n2t.net/addgene:60629; RRID: Addgene_60629) [54], plated on mouse collagen I-coated plates in differentiation medium and cultured for 28 days. Medium was changed daily. 96 h before experiments, the medium was changed with a differentiation medium without horse serum and containing 5% FBS (GIBCO, Cat. #26050088).

### Western Blot

USCs and USC-SkMCs cells were lysed with 100 µL of lysis buffer (50 mM Tris-HCl pH 7.4, sodium dodecyl sulphate (Sigma-Aldrich, Cat. #11667289001), 0.5%, 5 mM EDTA (Sigma-Aldrich, Cat. #E9884), 10 µL of protease inhibitors cocktail (PIC, Millipore, Cat. 539133) and collected in a 1.5 ml tube. Lysates were boiled at 96 °C for 5 min and then quantified with QuantiPro BCA Assay Kit (Sigma, Cat. SLBF3463). 40 µg of proteins were mixed with the right amount of Laemmli Sample Buffer (Bio-Rad, Cat. #1610747) and boiled. Then, samples were loaded on a 6–12% polyacrylamide-sodium dodecyl sulphate gel (according to the molecular weight of the protein of interest) for SDS-PAGE. Proteins were transferred onto nitrocellulose membrane, using Mini Transfer Packs or Midi Transfer Packs, with Trans-Blot® Turbo ^TM^ (Bio-Rad, Cat. #1704150) according to manufacturer’s instructions (Bio-Rad). The membranes were blocked in 5% skim milk (Sigma, Cat. 70166) for 45 min at room temperature (RT). Subsequently, membranes were incubated with indicated primary antibody, overnight at 4 °C. Primary antibodies used are listed in Supplementary Table 1, anti- β-Actin was used to normalize protein loading. Goat anti-mouse IgG (H + L) horseradish peroxidase-conjugated secondary antibody (Bio-Rad, 1:5000; Cat. 170-6516) and Goat anti-rabbit IgG (H + L) horseradish peroxidase-conjugated secondary antibody (Bio-Rad, 1:5000; Cat. 170-6515) were used as secondary antibodies. Detection was carried out with SuperSignal^TM^ West Pico/femto PLUS Chemiluminescent Substrate (Thermo Scientific Cat. #34580), based on the chemiluminescence of luminol and developed using ChemiDoc^TM^ Imaging System (Bio-Rad Cat. #12003153). Full-length uncropped original western blots used in the manuscript are provided as a single Original Data file.

### Immunofluorescence (IF)

USCs and USC-SkMCs cells were grown onto 13 mm glass coverslips for 48 h. Immunofluorescence was done as follows. Cells were fixed in 4% paraformaldehyde (Sigma Aldrich, Cat. # P6148) and 4% sucrose (Sigma Aldrich, Cat. #S0389), permeabilized (7 min in 0.1% Triton X-100 in phosphate-buffered saline (PBS), blocked in 0.1% gelatin, and immunoprobed with an appropriate primary antibody (Supplementary Table 1) overnight at 4 °C. After 3 times washing in PBS, Alexa-conjugated secondary antibodies (1:500) was applied for 1 h at RT. Secondary antibodies were Alexa Fluor 488 anti-mouse IgG (ThermoFisher, Cat. #A-11001), Alexa Fluor 488 anti-rabbit IgG (ThermoFisher, Cat. #A-11034), and Alexa Fluor 546 donkey anti-goat IgG (ThermoFisher, Cat. #A-11056). For Phalloidin staining, Phalloidin (Invitrogen, A30106) was incubated (1:400) together with a secondary antibody. After 3 times washing in PBS, nuclei were counter-stained with 4′,6-diamidino-2-phenylindole (DAPI, Sigma-Aldrich Cat. #D8417).

### Quantitative fluorescence image analysis

Images were acquired using a Leica SP8 LIGHTNING Confocal Microscope imaging system and a Leica TCS SP8 DIVE Multiphoton Microscope. Images were acquired under nonsaturating conditions and analysed with Fiji ImageJ 1.52p software. Data are expressed as fold change relative to control.

### RNA isolation and qPCR

Total RNA was isolated by Trizol (ThermoFisher Cat. #15596026) from USCs and SkMCs. The amount and purity of total RNA were quantified at the spectrophotometer (Nanodrop, Thermo Fisher) by measuring the optical density at 260 and 280 nm. 1 μg of total RNA was reverse-transcribed using a high-capacity SensiFAST™ cDNA Synthesis Kit (Bioline Cat. # BIO-65054) according to the manufacturer’s instructions. For quantitative polymerase chain reaction (qPCR) SensiFAST SYBR No-ROX kit (Bioline, Cat. #BIO-98020) and specific primers (Supplementary Table 2) were used. Glyceraldehyde-3-phosphate dehydrogenase (GAPDH) was the endogenous control.

### Single cell gel electrophoresis (comet assay)

For single-cell DNA damage analysis, we performed a comet assay as previously described [55]. Briefly, cells were cultured in a 24-wells plate, irradiated with UVB (40 mJ/cm^2^) for 35 s, and then, cells were left to recover for 6 h. Cells were mechanically detached, centrifuged, resuspended in 1 mL of cold PBS, and incubated on ice. Then, 250 μl cell suspension was mixed with 1 ml of melted low-melting agarose (Fisher Molecular Biology, Cat. #10377033) and transferred on agarose-coated microscope slides, incubated in lysis buffer overnight at 4 °C and then with the electrophoresis buffer for 30 min at 4 °C. Electrophoresis was performed using Comet Assay Tank (Cleaver Scientific, Rugby, UK) filled with 1 L of electrophoresis buffer and run for 30 min at 21 V and 400 mA. Samples were washed with dH_2_O for 5 min and stained with propidium iodide (PI) 10 μg/mL (Immunological Sciences, Cat. #IS-7715) for 20 min at RT in the dark. After the last wash with dH_2_O for 5 min, pictures were taken using a fluorescence microscope (DS5500B, Leica, Wetzlar, Germany), and quantification of the tail DNA percentage was performed using the automated CometScore 2.0 software (TrikTek, Berlin, Germany).

### Flow cytometry analysis

For surface markers analysis, cells were detached, centrifuged (900 *g*, 5 min) and incubated 1 h at 4 °C with CD146 Monoclonal Antibody (P1H12; PE; eBioscience™ Cat. #12-1469-42) and CD90 Monoclonal Antibody (5E10; FITC; eBioscience™ Cat. #11-0909-42). For the intracellular staining, USCs were fixed with PAF 4% 15 min at RT and permeabilized with PBS 1 × 0.3% Triton X-100 0.5% BSA, at RT for 1 h. Antibody staining was performed in PBS 1x with the following antibodies: OCT3/4 Monoclonal Antibody (EM92; Alexa Fluor™ 488, eBioscience™ Cat. # 53-5841-82) and SOX_2_ Monoclonal Antibody (Btjce; Alexa Fluor™ 488, eBioscience™ Cat. # 53-9811-82).

Cell cycle analysis was performed by flow cytometry. 150.000 USCs were fixed in 70% ethanol for 1 h at −20 °C. Then, cells were washed with PBS, resuspended in 200 μl propidium iodide buffer (3.4 mM trisodium citrate, 9.65 mM sodium chloride, and 0.003% tergitol), 25 μl RNasi A (10 ng/ml; Fisher Molecular Biology, Cat. #FS-RT-6600), and 10 μl propidium iodide (1 mg/ml) and incubated 15 min at 37 °C protected from light.

For annexinV/Propidium iodide (PI) staining cells were incubated with Annexin V Recombinant Protein 1x (FITC; eBioscience™, Cat. # BMS306FI-100) and PI 1 mg/ml in Annexin V binding buffer (PBS 1x, Hepes 10 mM, NaCl 150 mm, CaCl_2_ 2.5 mM) 15 min at RT in the dark. Fluorescence was quantified using Attune NxT (Life Technologies) flow cytometry.

### Ca^2+^ imaging

USCs and USC-SkMCs, grown onto 24 mm round coverslips (3 × 10^4^ cell/coverslip), were loaded with 2.5 μM Fura-2/AM (Cat. No. F1201, Life Technologies) in the presence of 0.005% pluronic F-127 (Cat. No. P6867, Life Technologies) and 10 μM sulfinpyrazone (Cat. S9509, Sigma Aldrich) in KRB solution (125 mM NaCl, 5 mM KCl, 1 mM Na_3_PO_4_, 1 mM MgSO_4_, 5.5 mM glucose, 20 mM HEPES, pH 7.4) supplemented with 2 mM CaCl_2_. After loading (30 min in the dark), cells were washed once with KRB solution and allowed to de-esterify for 30 min. After this, the coverslips were mounted in an acquisition chamber and placed on the stage of a Leica DM6000B epifluorescence microscope equipped with a S Fluor 40 × /1.3 objective. Cells were alternatively excited at 340/380 nm by the monochromator Polichrome V (Till Photonics, Munich, Germany), and the fluorescent signal was collected by a Hamamatsu cooled CCD camera through a bandpass 510/20 nm filter. The fluorescent signals were captured by MetaFluor software (Molecular Devices, Sunnyvale, CA, USA). The cells were stimulated with 200 μM ATP (Sigma Aldrich, Cat. #A6419) to detect cytosolic Ca^2+^ dynamics. Separate experiments were performed to measure SOCE (store-operated calcium entry). Changes in cytosolic Ca^2+^ were monitored upon depletion of the intracellular Ca^2+^ stores. Experiments were carried out during the exposure of the cells to the Ca^2+^-free solution. In the absence of Ca^2+^, the intracellular Ca^2+^ stores were depleted by tert-Butylhydroquinone (TBHQ, 50 µM; Sigma-Aldrich Cat. #11294) treatment. Re-addition of 2 mM Ca^2+^ allowed assessment of the SOCE. Baseline values are expressed as mean ± SEM of 340/380 Fura-2 ratio values (referred to as “Fura ratio”). For comparison of Ca^2+^ dynamics, measured as an amplitude of Ca^2+^ increase from the baseline level, Fura-2 ratio values were normalized using the formula (F_i_-F_0_)/F_0_ (referred to as “Normalized (Norm.) Fura Ratio”). For mitochondrial calcium dynamics assessment, mt-fura-2.3, a modified version of mt-fura-2, was used [26], and the same protocol described for Fura 2AM was followed.

### Endoplasmic reticulum Ca^2+^ imaging

ER Ca^2+^ dynamics were monitored with ER-GAP3 (AddGene, Cat. #78118), a genetically encoded Ca^2+^ sensor, targeted to the ER lumen (referred to as GAP3) [56]. 48 h post-transfection, expression of GAP3 was checked, and ER calcium dynamics were monitored. Coverslips were mounted in a chamber in KRB solution and placed on the stage of the microscope. Cells were alternately excited at 405 and 470 nm, and the fluorescent signal was acquired using a 510/20 nm bandpass filter. After recording basal signal for 20 s, KRB solution was removed and replaced with a Ca^2+^-free solution (KRB + 500 µM EGTA). After allowing the signal to stabilize for an additional 20 s, cells were stimulated with 100 µM ATP and 100 µM TBHQ, and the response was recorded for 200 s.

### Mitochondrial superoxide determination

For mitochondrial superoxide evaluation, cells were grown onto 13 mm glass coverslips for 48 h. Cells were washed 3 times with HBSS and incubated with 2 µM MitoSoxTM Red (Cat. No. M36008, Thermo Fisher Scientific) in complete media at 37 °C in the dark for 15 min. Then, cells were fixed in 4% paraformaldehyde and 4% sucrose and mounted for acquisition at confocal microscope with 40X magnification (Leica TCS SP8 LIGHTNING confocal laser scanning microscope).

### Contraction assay

USC-SkMCs-Ctr and USC-SkMCs-ATM-KO (400.000 cells/mL) were embedded in collagen gel (home-made from mouse tail, final concentration of 8 mg/mL) seeded in triplicate for each condition in 24-well plate and 96-well plate. After solidification, the gels were incubated in DMEM (Euroclone, Cat. #ECM0728L) with 5% FBS with or without acetylcholine (Sigma Aldrich, Cat. #A6625 (100 μM). Cells were left to grow, and the area of the collagen disk was measured from 1 h to 72 h after cells reached the confluence. Images of cells in the 96 well-plate were acquired using the Incucyte® Live-Cell Analysis System (Sartorius) and analysed by ImageJ software.

### Statistical analysis

Statistical analysis was performed with GraphPad Prism software (GraphPad Software Inc., La Jolla, CA). A two-tailed unpaired Student’s t-test was used to compare the two samples. To compare three or more samples, one-way ANOVA was used, unless otherwise specified. The data are expressed as the mean ± SEM of ‘n’ independent experiments performed in triplicate, as detailed in the figure legends, and were considered significant at *p* < 0.05.

## Supplementary information

Supplementary figures

Supplementary table 1

Supplementary Table 2

Original data

## Data Availability

The data supporting the findings of this study are available from the corresponding author upon reasonable request.

## References

[1] Lee JH, Paull TT. Cellular functions of the protein kinase ATM and their relevance to human disease. Nat Rev Mol Cell Biol. 2021;22:796–814. 34429537 10.1038/s41580-021-00394-2

[2] Amirifar P, Ranjouri MR, Lavin M, Abolhassani H, Yazdani R, Aghamohammadi A. Ataxia-telangiectasia: epidemiology, pathogenesis, clinical phenotype, diagnosis, prognosis and management. Expert Rev Clin Immunol. 2020;16:859–71. 32791865 10.1080/1744666X.2020.1810570

[3] Lavin MF. Ataxia-telangiectasia: from a rare disorder to a paradigm for cell signalling and cancer. Nat Rev Mol Cell Biol. 2008;9:759–69. 18813293 10.1038/nrm2514

[4] McKinnon PJ. ATM and the molecular pathogenesis of Ataxia Telangiectasia. Annu Rev Pathol Mech Dis. 2012;7:303–21. 10.1146/annurev-pathol-011811-132509 22035194

[5] Paull TT, Lee JH. The Mre11/Rad50/Nbs1 complex and its role as a DNA double-strand break sensor for ATM. Cell Cycle. 2005;4:737–40. 15908798 10.4161/cc.4.6.1715

[6] Bakkenist CJ, Kastan MB. DNA damage activates ATM through intermolecular autophosphorylation and dimer dissociation. Nat Gennaio. 2003;421:499–506. 10.1038/nature01368 12556884

[7] Lee Y, Chong MJ, McKinnon PJ. *Ataxia Telangiectasia Mutated* -dependent apoptosis after genotoxic stress in the developing nervous system is determined by cellular differentiation status. J Neurosci. 2001;21:6687–93. 11517258 10.1523/JNEUROSCI.21-17-06687.2001 PMC6763074

[8] Zhang Y, Lee JH, Paull TT, Gehrke S, D’Alessandro A, Dou Q, et al. Mitochondrial redox sensing by the kinase ATM maintains cellular antioxidant capacity. Sci Signal. 2018;11:eaaq0702. 29991649 10.1126/scisignal.aaq0702 PMC6042875

[9] Xie X, Zhang Y, Wang Z, Wang S, Jiang X, Cui H, et al. ATM at the crossroads of reactive oxygen species and autophagy. Int J Biol Sci. 2021;17:3080–90. 34421351 10.7150/ijbs.63963 PMC8375236

[10] Lee JH. Oxidative stress and the multifaceted roles of ATM in maintaining cellular redox homeostasis. Redox Biol. 2024;75:103269. 39018798 10.1016/j.redox.2024.103269 PMC11301354

[11] Ambrose M, Gatti RA. Pathogenesis of ataxia-telangiectasia: the next generation of ATM functions. Blood. 2013;121:4036–45. 23440242 10.1182/blood-2012-09-456897 PMC3709651

[12] Sullivan KD, Palaniappan VV, Espinosa JM. ATM regulates cell fate choice upon p53 activation by modulating mitochondrial turnover and ROS levels. Cell Cycle. 2015;14:56–63. 25483068 10.4161/15384101.2014.973330 PMC4614823

[13] Rothblum-Oviatt C, Wright J, Lefton-Greif MA, McGrath-Morrow SA, Crawford TO, Lederman HM. Ataxia telangiectasia: a review. Orphanet J Rare Dis. 2016;11:159. 27884168 10.1186/s13023-016-0543-7 PMC5123280

[14] Yeo AJ, Chong KL, Gatei M, Zou D, Stewart R, Withey S, et al. Impaired endoplasmic reticulum-mitochondrial signaling in ataxia-telangiectasia. iScience. 2021;24:101972. 33437944 10.1016/j.isci.2020.101972 PMC7788243

[15] Kuo IY, Ehrlich BE. Signaling in muscle contraction. Cold Spring Harb Perspect Biol. 2015;7:a006023. 25646377 10.1101/cshperspect.a006023 PMC4315934

[16] Miterko LN, Lin T, Zhou J, Van Der Heijden ME, Beckinghausen J, White JJ, et al. Neuromodulation of the cerebellum rescues movement in a mouse model of ataxia. Nat Commun. 2021;12:1295. 33637754 10.1038/s41467-021-21417-8 PMC7910465

[17] Verhagen M, Alfen N, Pillen S, Weemaes C, Yntema J, Hiel J, et al. Neuromuscular abnormalities in Ataxia Telangiectasia: A clinical, electrophysiological and muscle ultrasound study. Neuropediatrics. 2007;38:117–21. 17985259 10.1055/s-2007-985899

[18] Focchi E, Cambria C, Pizzamiglio L, Murru L, Pelucchi S, D’Andrea L, et al. ATM rules neurodevelopment and glutamatergic transmission in the hippocampus but not in the cortex. Cell Death Dis. 2022;13:616. 35842432 10.1038/s41419-022-05038-7 PMC9288428

[19] Crawford TO, Mandir AS, Lefton-Greif MA, Goodman SN, Goodman BK, Sengul H, et al. Quantitative neurologic assessment of ataxia-telangiectasia. Neurology. 2000;54:1505–9. 10751267 10.1212/wnl.54.7.1505

[20] Yu P, Bosholm CC, Zhu H, Duan Z, Atala A, Zhang Y. Beyond waste: understanding urine’s potential in precision medicine. Trends Biotechnol. 2024;42:953–69. 38369434 10.1016/j.tibtech.2024.01.009 PMC11741143

[21] Sun Y, Zhao H, Yang S, Wang G, Zhu L, Sun C, et al. Urine-derived stem cells: Promising advancements and applications in regenerative medicine and beyond. Heliyon. 2024;10:e27306. 38509987 10.1016/j.heliyon.2024.e27306 PMC10951541

[22] Cavaleiro C, Afonso GJM, Oliveira PJ, Valero J, Mota SI, Ferreiro E. Urine-derived stem cells in neurological diseases: current state-of-the-art and future directions. Front Mol Neurosci. 2023;16:1229728. 37965041 10.3389/fnmol.2023.1229728 PMC10642248

[23] Talmon M, Massara E, Pruonto G, Quaregna M, Boccafoschi F, Riva B, et al. Characterization of a functional Ca2+ toolkit in urine-derived stem cells and derived skeletal muscle cells. Cell Calcium. 2022;103:102548. 35144096 10.1016/j.ceca.2022.102548

[24] Talmon M, Massara E, Quaregna M, De Battisti M, Boccafoschi F, Lecchi G, et al. Bitter taste receptor (TAS2R) 46 in human skeletal muscle: expression and activity. Front Pharm. 2023;14:1205651. 10.3389/fphar.2023.1205651 PMC10522851 37771728

[25] Rodríguez-Prados M, Rojo-Ruiz J, Aulestia FJ, García-Sancho J, Alonso MT. A new low-Ca2+ affinity GAP indicator to monitor high Ca2+ in organelles by luminescence. Cell Calcium. 2015;58:558–64. 26412347 10.1016/j.ceca.2015.09.002

[26] De Nadai A, Vajente N, Pendin D, Mattarei A. Mt-fura-2, a Ratiometric Mitochondria-Targeted Ca2+ Sensor. Determination of Spectroscopic Properties and Ca2+ Imaging Assays. In: et al., curatori. Mitochondrial Medicine. New York, NY: Springer US; 2021. p. 187–215. (Methods in Molecular Biology; vol. 2275). Disponibile su: https://link.springer.com/10.1007/978-1-0716-1262-0_12. 10.1007/978-1-0716-1262-0_12 34118039

[27] Lim D, Dematteis G, Tapella L, Genazzani AA, Calì T, Brini M, et al. Ca2+ handling at the mitochondria-ER contact sites in neurodegeneration. Cell Calcium. 2021;98:102453. 34399235 10.1016/j.ceca.2021.102453

[28] Andrisse S, Patel GD, Chen JE, Webber AM, Spears LD, Koehler RM, et al. ATM and GLUT1-S490 Phosphorylation regulate GLUT1 mediated transport in skeletal muscle. Planas JV, curatore. PLoS One 2013;8:e66027. 23776597 10.1371/journal.pone.0066027 PMC3679034

[29] Ching JK, Luebbert SH, Collins Iv RL, Zhang Z, Marupudi N, Banerjee S, et al. Ataxia telangiectasia mutated impacts insulin‐like growth factor 1 signalling in skeletal muscle. Exp Physiol. 2013;98:526–35. 22941977 10.1113/expphysiol.2012.066357 PMC3553258

[30] Nayler S, Gatei M, Kozlov S, Gatti R, Mar JC, Wells CA, et al. Induced Pluripotent stem cells from Ataxia-Telangiectasia recapitulate the cellular phenotype. Stem Cells Transl Med. 2012;1:523–35. 23197857 10.5966/sctm.2012-0024 PMC3659724

[31] Momčilović O, Choi S, Varum S, Bakkenist C, Schatten G, Navara C. Ionizing radiation induces Ataxia Telangiectasia mutated-dependent checkpoint signaling and G2 But Not G1 cell cycle arrest in pluripotent human embryonic stem cells. Stem Cells. 2009;27:1822–35. 19544417 10.1002/stem.123 PMC3340923

[32] Valentin-Vega YA, MacLean KH, Tait-Mulder J, Milasta S, Steeves M, Dorsey FC, et al. Mitochondrial dysfunction in ataxia-telangiectasia. Blood. 2012;119:1490–500. 22144182 10.1182/blood-2011-08-373639 PMC3286212

[33] Ambrose M, Goldstine JV, Gatti RA. Intrinsic mitochondrial dysfunction in ATM-deficient lymphoblastoid cells. Hum Mol Genet. 2007;16:2154–64. 17606465 10.1093/hmg/ddm166

[34] Eaton JS, Lin ZP, Sartorelli AC, Bonawitz ND, Shadel GS. Ataxia-telangiectasia mutated kinase regulates ribonucleotide reductase and mitochondrial homeostasis. J Clin Invest. 2007;117:2723–34. 17786248 10.1172/JCI31604 PMC1952633

[35] Rossi A, Pizzo P, Filadi R. Calcium, mitochondria and cell metabolism: A functional triangle in bioenergetics. Biochim Biophys Acta BBA - Mol Cell Res. 2019;1866:1068–78. 10.1016/j.bbamcr.2018.10.016 30982525

[36] Tassinari V, De Gennaro V, La Sala G, Marazziti D, Bolasco G, Aguanno S, et al. Atrophy, oxidative switching and ultrastructural defects in skeletal muscle of Ataxia Telangiectasia mouse model. J Cell Sci. 2019;132:223008. 10.1242/jcs.223008 30745336

[37] Jeong I, Patel AY, Zhang Z, Patil PB, Nadella ST, Nair S, et al. Role of ataxia telangiectasia mutated in insulin signalling of muscle‐derived cell lines and mouse soleus. Acta Physiol April. 2010;198:465–75. 10.1111/j.1748-1716.2009.02069.x PMC2875195 20003097

[38] Yang DQ, Kastan MB. Participation of ATM in insulin signalling through phosphorylation of eIF-4E-binding protein 1. Nat Cell Biol. 2000;2:893–8. 11146653 10.1038/35046542

[39] Culenova M, Nicodemou A, Novakova ZV, Debreova M, Smolinská V, Bernatova S, et al. Isolation, culture and comprehensive characterization of biological properties of human urine-derived stem cells. Int J Mol Sci. 2021;22:12503. 34830384 10.3390/ijms222212503 PMC8624597

[40] Lang R, Liu G, Shi Y, Bharadwaj S, Leng X, Zhou X, et al. Self-renewal and differentiation capacity of urine-derived stem cells after urine preservation for 24 hours. PLoS One 2013;8:e53980. 23349776 10.1371/journal.pone.0053980 PMC3548815

[41] Falzarano MS, Ferlini A. Urinary stem cells as tools to study genetic disease: overview of the literature. J Clin Med. 2019;8:627. 31071994 10.3390/jcm8050627 PMC6572423

[42] Benda C, Zhou T, Wang X, Tian W, Grillari J, Tse HF, et al. Urine as a Source of Stem Cells. In: Weyand B, Dominici M, Hass R, Jacobs R, Kasper C, curatori. Mesenchymal Stem Cells - Basics and Clinical Application I. Berlin, Heidelberg: Springer Berlin Heidelberg; 2012. p. 19–32. (Advances in Biochemical Engineering/Biotechnology; vol. 129). Disponibile su: https://link.springer.com/10.1007/10_2012_157. 10.1007/10_2012_157 23038280

[43] Liu W, Zhang P, Tan J, Lin Y. Differentiation of urine-derived induced pluripotent stem cells to neurons, astrocytes, and microvascular endothelial cells from a diabetic patient. Cell Reprogram. 2020;22:147–55. 32207986 10.1089/cell.2019.0088

[44] Xu G, Wu F, Gu X, Zhang J, You K, Chen Y, et al. Direct conversion of human urine cells to neurons by small molecules. Sci Rep. 2019;9:16707. 31723223 10.1038/s41598-019-53007-6 PMC6854089

[45] Chen W, Xie M, Yang B, Bharadwaj S, Song L, Liu G, et al. Skeletal myogenic differentiation of human urine-derived cells as a potential source for skeletal muscle regeneration: Skeletal myogenic differentiation of hUSCs. J Tissue Eng Regen Med. 2017;11:334–41. 24945524 10.1002/term.1914

[46] Lazzeri E, Ronconi E, Angelotti ML, Peired A, Mazzinghi B, Becherucci F, et al. Human urine-derived renal progenitors for personalized modeling of genetic kidney disorders. J Am Soc Nephrol. 2015;26:1961–74. 25568173 10.1681/ASN.2014010057 PMC4520157

[47] Guo D, Wu F, Liu H, Gao G, Kou S, Yang F, et al. Generation of non-integrated induced pluripotent stem cells from a 23-year-old male with multiple endocrine neoplasia type 1 syndrome. Stem Cell Res. 2017;18:70–2. 28395810 10.1016/j.scr.2016.12.002

[48] Zhang Y, Niu X, Dong X, Wang Y, Li H. Bioglass enhanced wound healing ability of urine‐derived stem cells through promoting paracrine effects between stem cells and recipient cells. J Tissue Eng Regen Med. marzo 2018;12. https://onlinelibrary.wiley.com/doi/10.1002/term.2587. 10.1002/term.2587 29024443

[49] Slaats GG, Braun F, Hoehne M, Frech LE, Blomberg L, Benzing T, et al. Urine-derived cells: a promising diagnostic tool in Fabry disease patients. Sci Rep. 2018;8:11042. 30038331 10.1038/s41598-018-29240-w PMC6056427

[50] Takizawa H, Sato M, Aoki Y. Exon skipping in directly reprogrammed myotubes obtained from human urine-derived cells. J Vis Exp. 2020;159:60840. 10.3791/60840 32449741

[51] Falzarano MS, D’Amario D, Siracusano A, Massetti M, Amodeo A, La Neve F, et al. Duchenne muscular dystrophy myogenic cells from urine-derived stem cells recapitulate the Dystrophin genotype and phenotype. Hum Gene Ther. 2016;27:772–83. 27530229 10.1089/hum.2016.079

[52] Zhang QJ, Lin X, Li JJ, Lu YQ, Guo XX, Dong EL, et al. Application of urine cells in drug intervention for spinal muscular atrophy. Exp Ther Med. 2017;14:1993–8. 28962115 10.3892/etm.2017.4791 PMC5609093

[53] Falzarano MS, Rossi R, Grilli A, Fang M, Osman H, Sabatelli P, et al. Urine-derived stem cells Express 571 neuromuscular disorders causing genes, making them a potential in vitro model for rare genetic diseases. Front Physiol. 2021;12:716471. 34744760 10.3389/fphys.2021.716471 PMC8565768

[54] Kabadi AM, Thakore PI, Vockley CM, Ousterout DG, Gibson TM, Guilak F, et al. Enhanced MyoD-induced transdifferentiation to a myogenic lineage by fusion to a potent transactivation domain. ACS Synth Biol. 2015;4:689–99. 25494287 10.1021/sb500322u PMC4475448

[55] Clementi E, Garajova Z, Markkanen E. Measuring DNA damage using the alkaline comet assay in cultured cells. BIO-Protoc. 2021;11.: https://bio-protocol.org/e4119. 10.21769/BioProtoc.4119 PMC8413625 34541038

[56] Navas-Navarro P, Rojo-Ruiz J, Rodriguez-Prados M, Ganfornina MD, Looger LL, Alonso MT, et al. GFP-Aequorin protein sensor for ex vivo and in vivo imaging of Ca2+ dynamics in high-Ca2+ Organelles. Cell Chem Biol. 2016;23:738–45. 27291400 10.1016/j.chembiol.2016.05.010

