# Supplementary material for: ATM knock out alters calcium signalling and augments contraction in skeletal muscle cells differentiated from human urine-derived stem cells
Source: Cell Death Discov. 2025 Apr 15;11:177. doi: 10.1038/s41420-025-02485-x (PMC12000312; doi:10.1038/s41420-025-02485-x)
Supplement: Supplementary file 1 — Supplementary figures [file 41420_2025_2485_MOESM1_ESM.docx]

**SUPPLEMENTARY FIGURES**

**Supplementary fig 1. USC-ATM-KO characterization.** Representative Western blot and densitometric analysis of ATM expression in both USC-Ctr and ATM-KO. Data are mean±SEM (n=6 independent experiments) of the % of the Ctr. ***p<0.0001 vs Ctr.


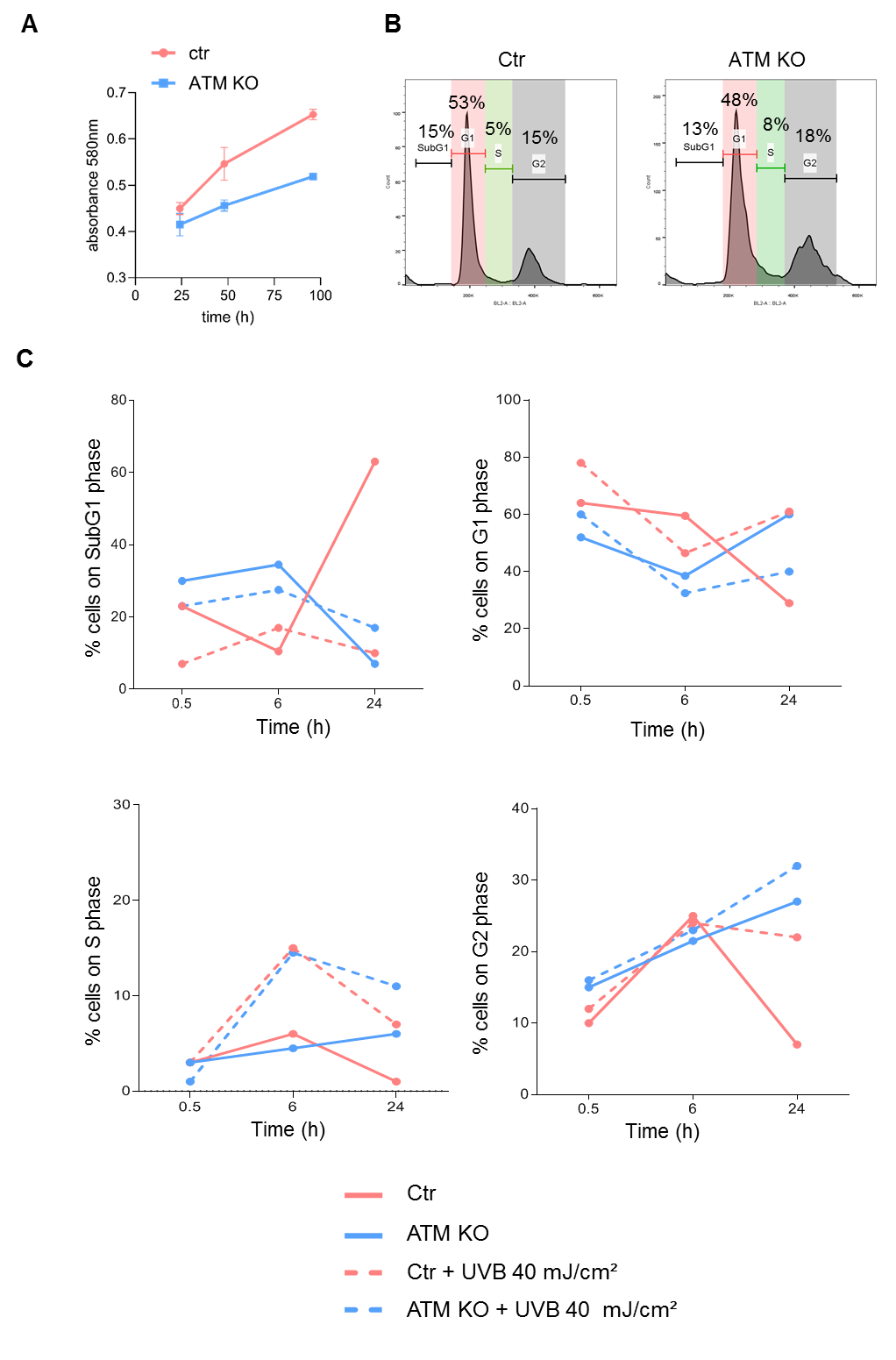


**Supplementary fig. 2. USCs proliferation and cell cycle analysis.** A) Crystal violet proliferation assay. Absorbance (580 nm) was analyzed 20, 40 and 80 hours after cells seeding. Ctr, USCs wild type; ATM-KO, USCs knock out for ATM protein. ***p*<0.01 and *****p*<0.0001 *vs* USCs-Ctr. B) Representative histograms of cell cycle analysis. Cells were stained with propidium iodide (PI) and the fluorescence analyzed by cytofluorimeter (channel BL2-A). Data are expressed as percentage of positive cells. C) Cells were stimulated with UVB 40 mJ/cm^2^ and, after 0.5, 6 and 24 h of recovery, stained with PI and analysed at the cytofluorimeter. Data are expressed as % of PI positive cells in the different conditions (Ctr, ATM-KO ± UVB stimulus) in the several cell cycles phases (SubG1, G1, S and G2).

**Supplementary fig 3. USC-SkMC-ATM-KO characterization.** Representative Western blot and densitometric analysis of ATM expression in skeletal muscle cells derived from both USC-Ctr (SkMC-Ctr) and ATM-KO (SkMC-ATM-KO). Data are mean±SEM (n=4 independent experiments) of the % of the Ctr. ***p<0.05 vs Ctr.

**Supplementary table 1.** List of primary antibodies used for western blot and immunofluorescence.

**Supplementary table 2.** List of primers for qPCR.
