# Supplementary material for: ATM knock out alters calcium signalling and augments contraction in skeletal muscle cells differentiated from human urine-derived stem cells
Source: Cell Death Discov. 2025 Apr 15;11:177. doi: 10.1038/s41420-025-02485-x (PMC12000312; doi:10.1038/s41420-025-02485-x)
Supplement: Supplementary file 4 — Original data [file 41420_2025_2485_MOESM4_ESM.pdf]

ORIGINAL DATA

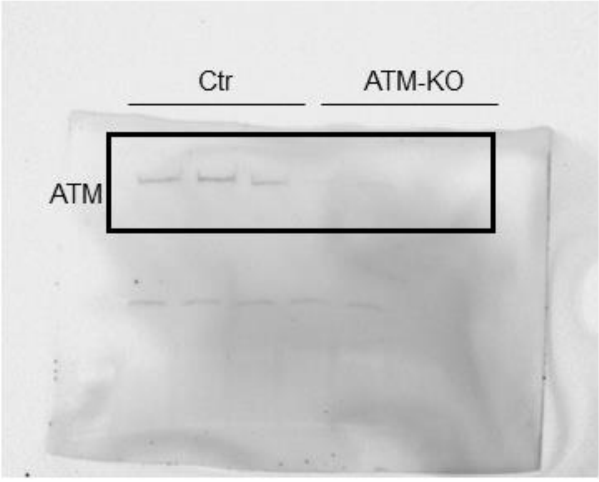

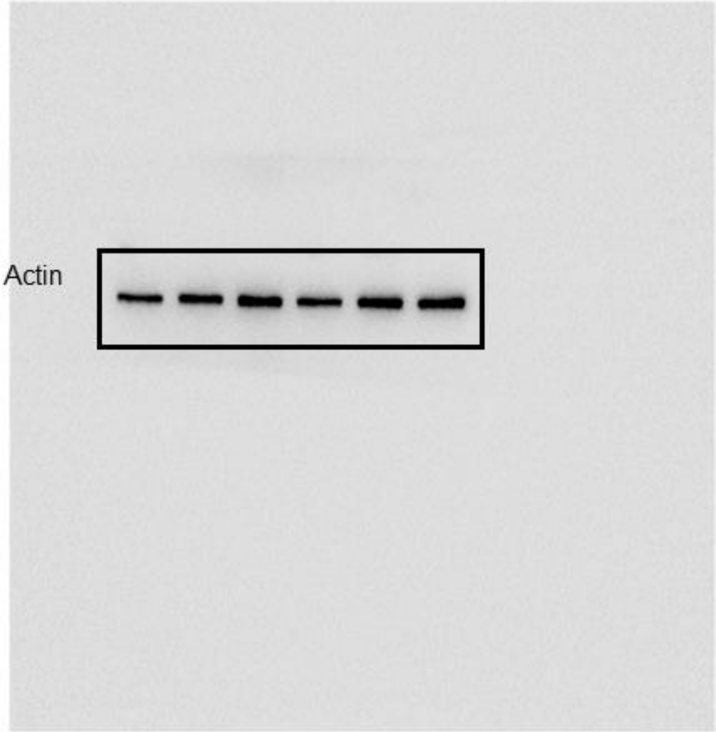

Original images of representative Western of ATM expression in USC-Ctr and USC-ATM-KO (main Fig 1B).

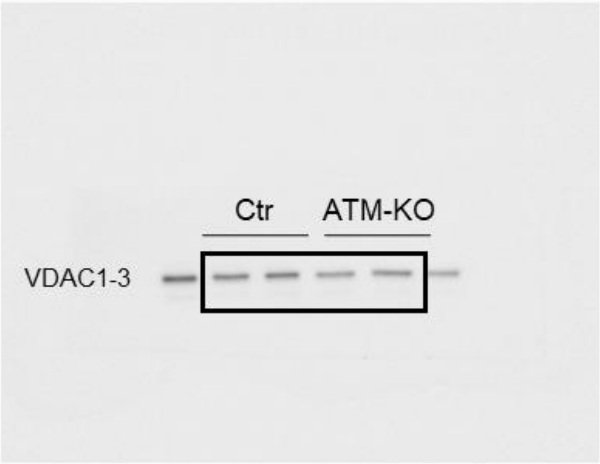
Ctr
ATM-KO
VDAC1-3

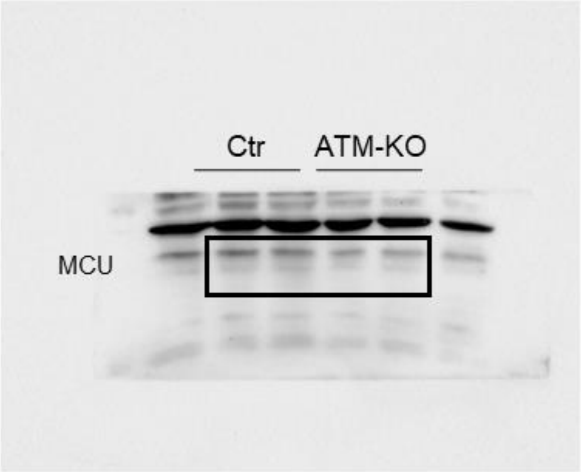
Ctr
ATM-KO
MCU

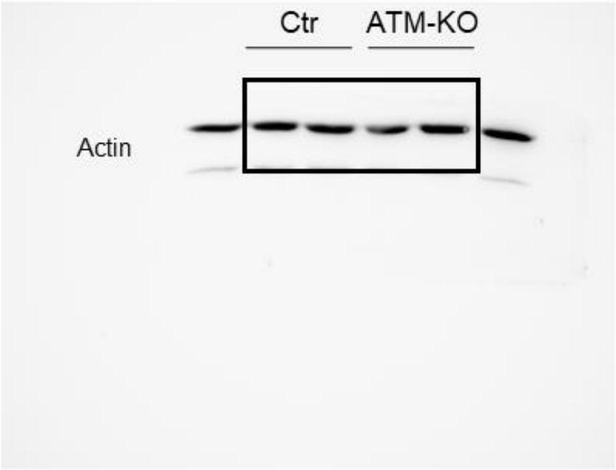
Ctr
ATM-KO
Actin

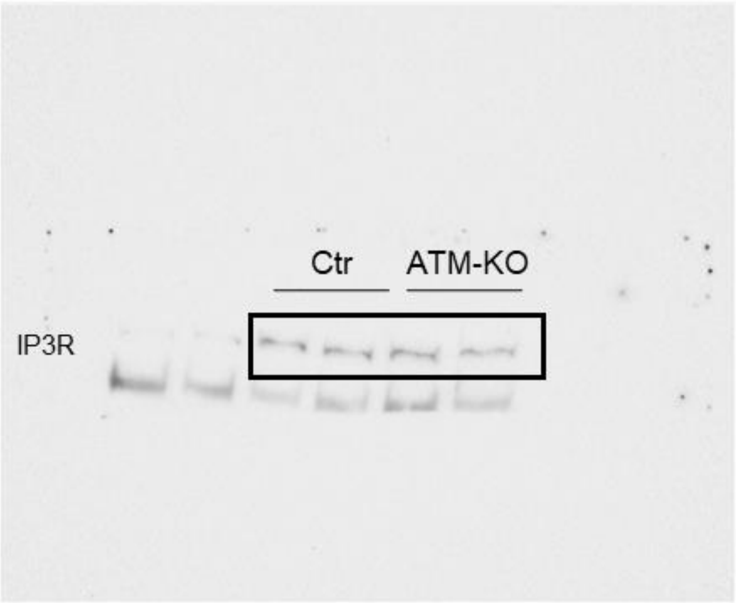

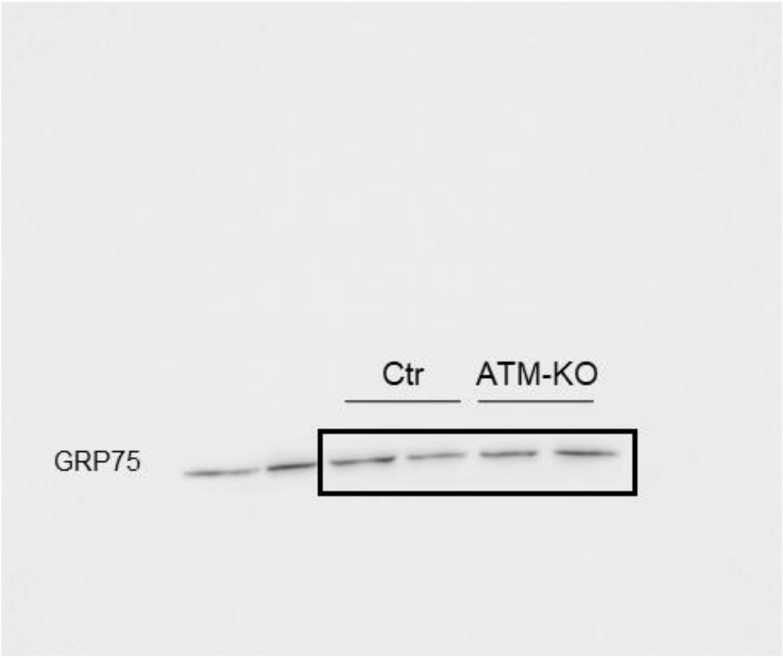

Original images of representative Western blot of IP3R, GRP75, VDAC1-3 and MCU expression in USC-Ctr and USC-ATM-KO (main Fig. 4)

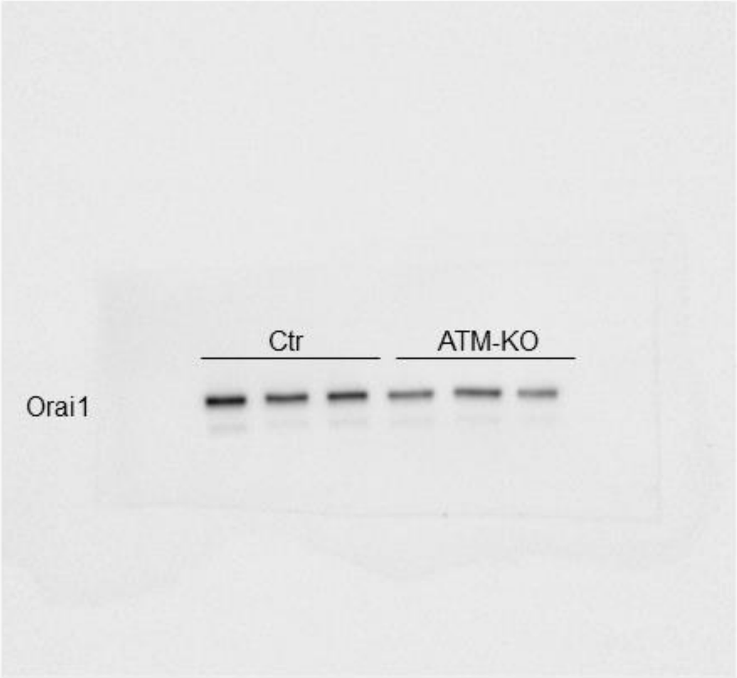
Ctr
ATM-KO
Orai1

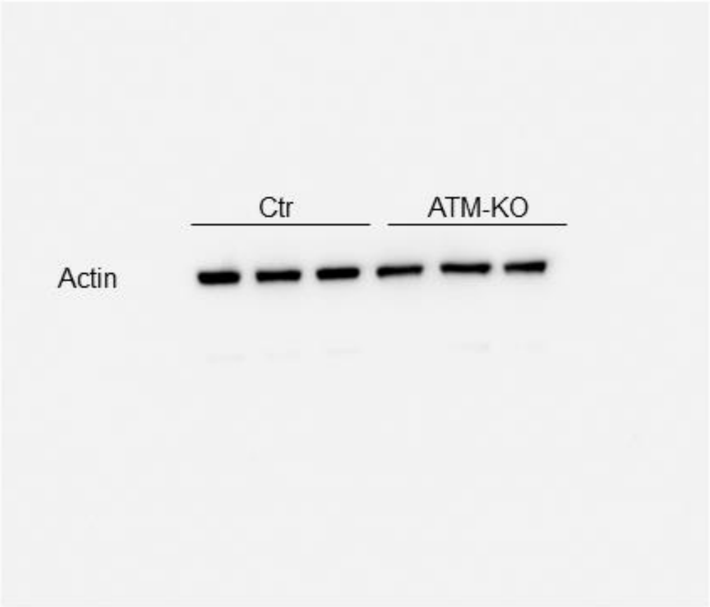
Ctr
ATM-KO
Actin

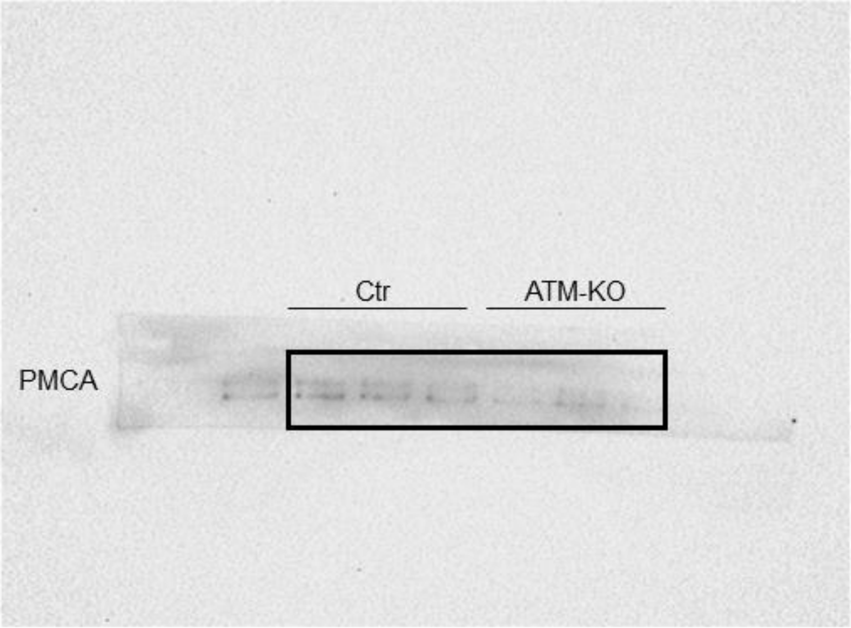

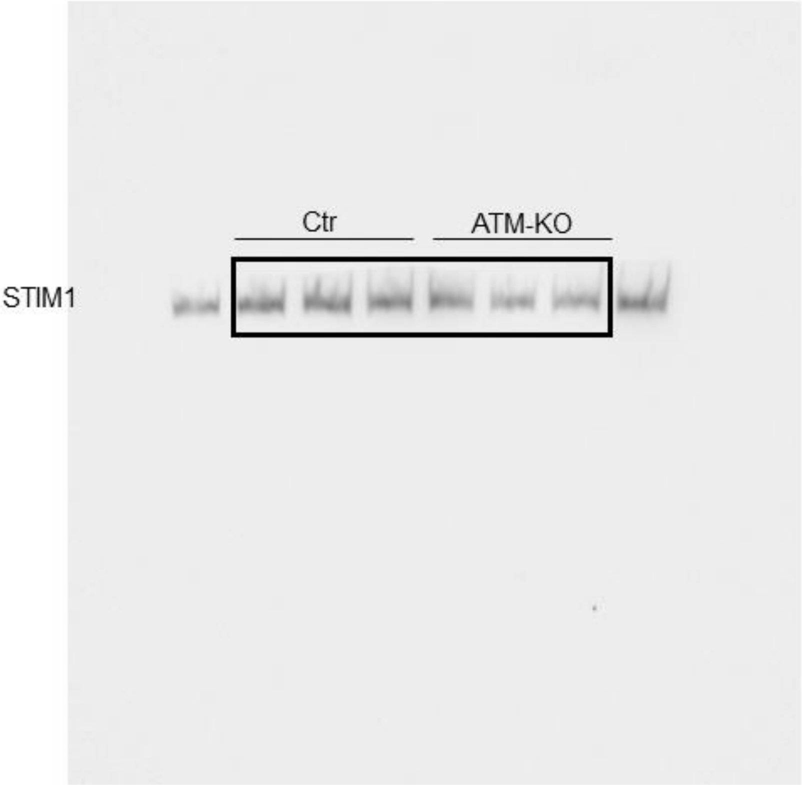

Original images of representative Western blots of PMCA, STIM, ORA1 expression in skeletal muscle cells derived from bot USC-Ctr (SkMC-Ctr) and ATM-KO (SkMC-ATM-KO) (Main Fig. 7A)

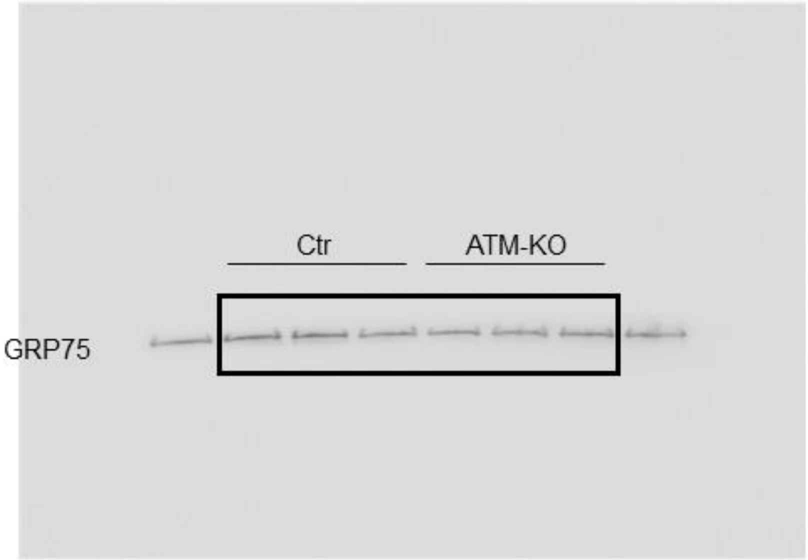
Ctr
ATM-KO
GRP75

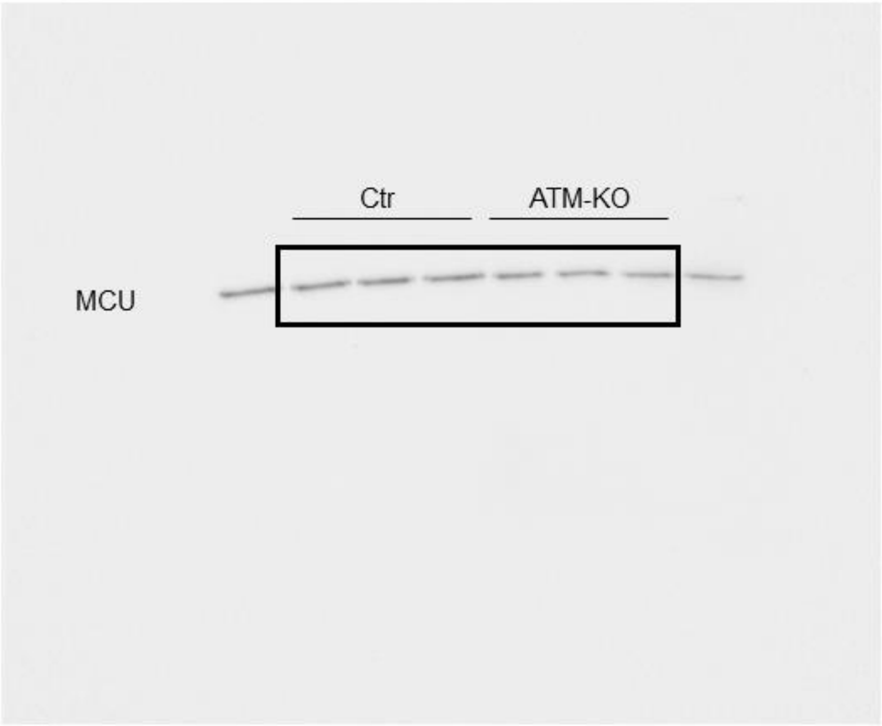
Ctr
ATM-KO
MCU

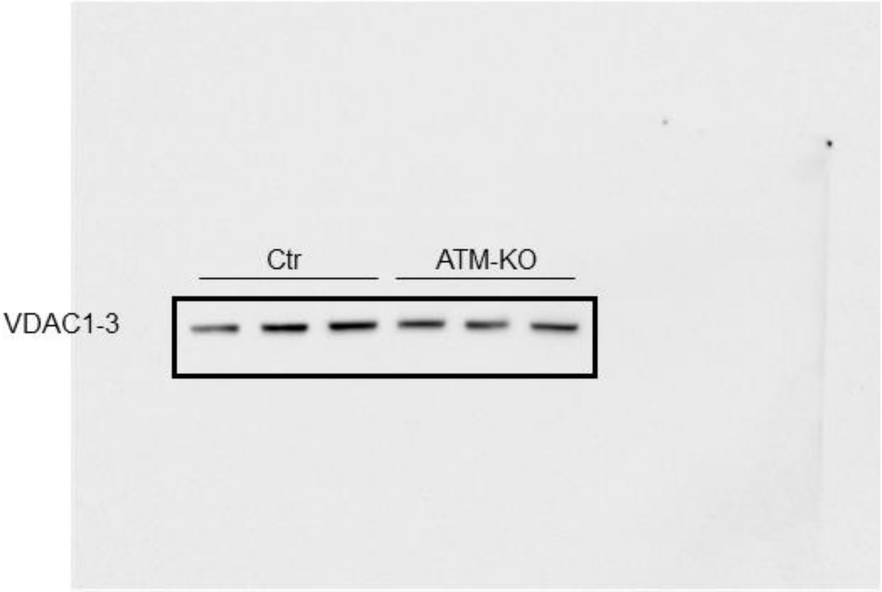

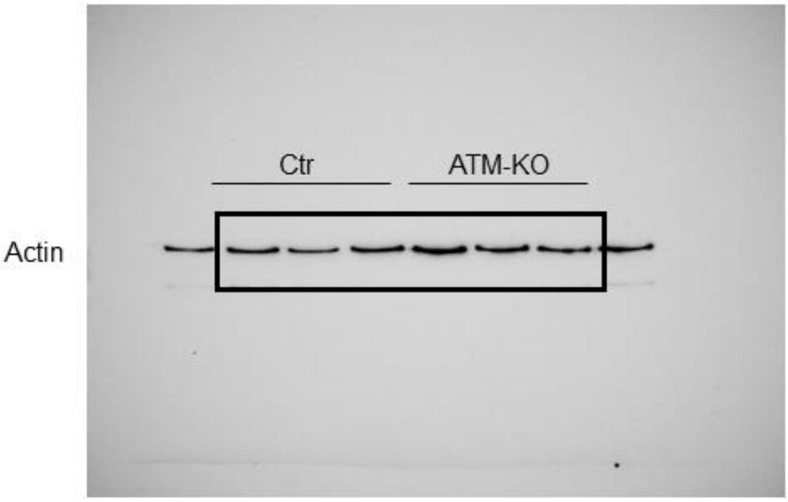

Original images of representative Western blots of GPR75, MCU, VDAC1-3 expression in skeletal muscle cells derived from bot USC-Ctr (SkMC-Ctr) and ATM-KO (SkMC-ATM-KO) (Main Fig. 7B)

ATM

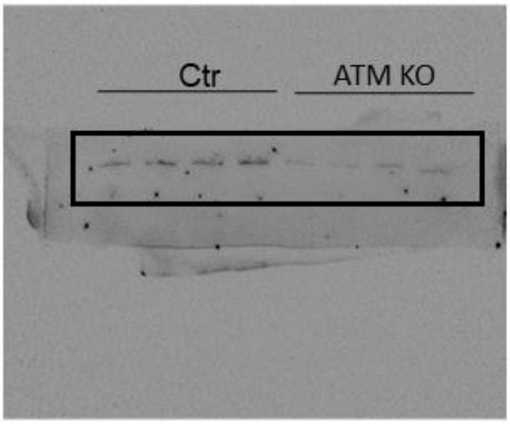

Actin

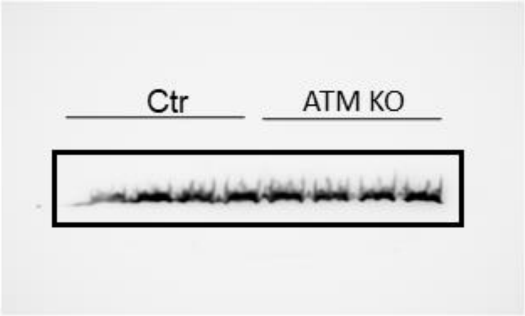

Original images of representative Western blots of ATM expression in skeletal muscle cells derived from bot USC-Ctr (SkMC-Ctr) and ATM-KO (SkMC-ATM-KO) (Supplementary Fig.2)
